# Barriers and Enablers to Using a Mobile App–Based Clinical Decision Support System in Managing Perioperative Adverse Events Among Anesthesia Providers: Cross-Sectional Survey in China

**DOI:** 10.2196/60304

**Published:** 2025-05-13

**Authors:** Xixia Feng, Peiyi Li, Renjie Zhao, Weimin Li, Tao Zhu, Xuechao Hao, Guo Chen

**Affiliations:** 1 Department of Anesthesiology West China Hospital Sichuan University Chengdu, Sichuan China; 2 Laboratory of Anesthesia and Critical Care Medicine, National-Local Joint Engineering Research Centre of Translational Medicine of Anesthesiology West China Hospital Sichuan University Chengdu, Sichuan China; 3 The Research Units of West China (2018RU012)-Chinese Academy of Medical Sciences West China Hospital Sichuan University Chengdu, Sichuan China; 4 West China School of Medicine Sichuan University Chengdu, Sichuan China; 5 Department of Respiratory and Critical Care Medicine West China Hospital Sichuan University Chengdu, Sichuan China; 6 Institute of Respiratory Health, Frontiers Science Center for Disease-related Molecular Network West China Hospital Sichuan University Chengdu, Sichuan China

**Keywords:** anesthesiologists, clinical decision support systems, mobile applications, nurse anesthetists, perioperative care, perioperative nursing, safety management

## Abstract

**Background:**

Perioperative adverse events (PAEs) pose a substantial global health burden, contributing to elevated morbidity, mortality, and health care expenditures. The adoption of clinical decision support systems (CDSS), particularly mobile-based solutions, offers a promising avenue to address these challenges. However, successful implementation hinges on understanding anesthesia providers’ knowledge, attitudes, and willingness to embrace such technologies.

**Objective:**

This study aimed to evaluate the knowledge, attitudes, and willingness of Chinese anesthesia professionals to adopt a mobile CDSS for PAE management, and to identify key factors influencing its implementation.

**Methods:**

A nationwide cross-sectional survey was conducted among anesthesia providers in China from September 5 to December 31, 2023. Participants included anesthesiologists and nurse anesthetists, who play pivotal roles in perioperative care. A 51-item questionnaire, structured around the Knowledge-Attitude-Practice (KAP) framework, was distributed via WeChat through professional anesthesia associations. The questionnaire covered four domains: (1) demographic characteristics, (2) knowledge assessment, (3) attitude evaluation, and (4) practice willingness. Multivariable regression analyses identified predictors of KAP outcomes, with sensitivity analyses focusing on nurse anesthetists.

**Results:**

The study included 2440 anesthesia professionals (2226 anesthesiologists and 214 nurse anesthetists). Overall, 87.3% (2130/2440) expressed willingness to adopt the CDSS, with 87.5% (1947/2226) of anesthesiologists and 85.5% (183/214) of nurse anesthetists showing readiness. However, only 39.2% (956/2440) were satisfied with existing incident management systems. Key findings indicated that higher knowledge scores were associated with female gender (coefficient=0.19, *P*=.003), advanced education, and lack of previous informatics experience (coefficient=0.29, *P*<.001). Nurse anesthetists scored lower than anesthesiologists (coefficient=–0.76, *P*<.001). Negative attitudes were more prevalent among older practitioners (coefficient=–0.13, *P*<.001), females (coefficient=–0.66, *P*<.001), nurse anesthetists (coefficient=–1.12, *P*=.003), and those without prior PAE exposure (coefficient=–0.97, *P*<.001). Higher willingness was observed among practitioners in Southwest China (coefficient=0.10, *P*=.048), those with positive attitudes (coefficient=0.06, *P*<.001), and those dissatisfied (coefficient=0.32, *P*<.001) or neutral (coefficient=0.11, *P*=.02) towards existing systems. Infrequent departmental incident discussions would reduce practice willingness (coefficient=–0.08, *P*=.01).

**Conclusions:**

This national study highlights a strong readiness among Chinese anesthesia professionals to adopt mobile CDSS for PAE management. However, critical barriers, including role-specific knowledge disparities and ineffective organizational communication, must be addressed to ensure successful implementation. Collaborative efforts among local authorities, health care facilities, anesthesia departments, and technology developers are essential to design and implement tailored strategies. Key recommendations include interdisciplinary training programs to enhance nurse anesthetists’ competencies, institution-level incentives to promote incident reporting, and user-centered CDSS designs that prioritize seamless integration into clinical workflows. These measures are vital for improving perioperative incident reporting systems and ultimately advancing the safety and outcomes of surgical patients.

## Introduction

Adverse events (AEs), defined as “harm caused by healthcare process rather than the patient’s underlying disease,” pose a global public health challenge and account for substantial mortality worldwide [1,2]. Recent statistics indicate that AEs are alarmingly common in health care settings, with low- and middle-income countries experiencing approximately 134 million AEs annually, contributing to around 2.6 million deaths [3]. Surgical care contributes significantly to this burden. Perioperative AEs (PAEs) directly affect 7 million patients annually [3,4], while recent data show that 4.2 million patients die within 30 days postoperatively worldwide, representing 7.7% of global mortality and ranking as the third leading cause of death, which underscores PAEs as a critical global health challenge.[5] The interplay of factors such as the complexity of surgical care, the coordination required among a large health care team, the rising volume of complex surgeries, and the rising prevalence of patients presenting with complex comorbidities contribute to the elevated incidence and risk of PAEs [6-9].

Many AEs (including PAEs) are preventable, and real-time monitoring along with early identification is crucial for enabling prompt responses and timely interventions [10-12]. However, current reporting systems remain hampered by underreporting, unclear investigative processes, and delayed responses [13-15]. Emerging information technologies like artificial intelligence and clinical decision support systems (CDSSs) show potential for real-time monitoring and rapid warning [16-20], as demonstrated in adverse drug event (ADE) management [19]. We previously proposed a mobile app–based CDSS for PAE management, integrating automated vital sign monitoring and predictive risk alerts to enable proactive interventions [21]. Accordingly, an automated and collaborative reporting mechanism was introduced to reveal concealed medical errors, thus cultivating a culture of transparency and proactive governance of PAEs [21].

The successful integration of health care technologies requires a comprehensive understanding of end-user needs, particularly those of health care providers operating at the human-technology interface in complex clinical settings [22-24]. In China, anesthesiologists are licensed physicians specializing in perioperative care, responsible for anesthesia planning, administration, and patient monitoring across the perioperative period [25]. They oversee the entire anesthesia process, including preoperative assessment, intraoperative management, and postoperative recovery. Nurse anesthetists support anesthesiologists by assisting in anesthesia administration and patient monitoring, especially during the maintenance phase [25,26]. Despite their crucial role in perioperative safety, anesthesia providers remain an understudied group. Existing studies on AE management have largely focused on ADEs rather than PAEs, with limited involvement of anesthesia professionals [27,28]. This study seeks to investigate anesthesia providers’ perspectives on PAE management and a mobile appbased CDSS in China, using a self-designed questionnaire. By addressing the specific needs and concerns of anesthesia providers, the study aims to lay the foundation for the development of a mobile app–based CDSS for PAE reporting and management.

## Methods

### Study Design and Participants

This nationwide cross-sectional survey in China was conducted by West China Hospital, a national medical center located in Chengdu, Sichuan Province [29], from September 5 to December 31, 2023. The study adhered to the CHERRIES (Checklist for Reporting Results of Internet E-Surveys) reporting guidelines [30].

Inclusion criteria were (1) participants must be anesthesia providers (certified registered anesthesiologists and nurse anesthetists), providing direct clinical care in both public and private health care facilities; (2) participants should be able to independently complete the questionnaire; (3) informed consent must be obtained; and (4) participants must have access to the internet and suitable devices for the web-based survey.

### Questionnaire Design and Development

The questionnaire was developed based on the knowledge, attitude, and practice (KAP) theory, which highlights the relationships among health behavior changes [31]. In addition to an extensive literature review pertaining to PAEs [23,32-37], we conducted a focus group interview with 10 stakeholders, including surgeons, anesthesia providers, epidemiologists, medical informaticists, health care policymakers, and information technicians from different health service systems, ensuring the relevance and comprehensiveness of the questionnaire items.

Internal consistency was assessed using Cronbach alpha, which yielded a value above 0.7, indicating satisfactory reliability [36]. Exploratory factor analysis showed a Kaiser-Meyer-Olkin value of 0.87, and the Bartlett test of sphericity was significant (*P*<.001) [38]. Factor analysis demonstrated that the cumulative contribution rate of the 6 factors with eigenvalues greater than 1 was determined to be 54.01, confirming good structural validity. Content validity was assessed by 5 experts from various fields (anesthesiology, nursing, statistics, health care policy, and administration) using a 4-point Likert scale (1=not relevant, 2=somewhat relevant, 3=quite relevant, and 4=highly relevant) [39]. The item-level content validity index was determined using the formula (the item-level content validity index=number of experts rating 3 or 4/total number of experts) [40]. Items with a content validity index greater than 0.8 were retained. After expert revisions, a pretest was conducted with 20 anesthesia professionals to further adjust and refine the questionnaire. The final version included 51 items divided into 4 sections (Multimedia Appendices 1 and 2).

### Sociodemographic Information

The first section of the survey gathered general information about participants, including their age, gender, professional role (anesthesiologist or nurse anesthetist), educational background, professional title, length of service in health care, and the city and hospital tier at which they were used. In China, public hospitals are categorized into 3 tiers based on their medical capabilities: tier 1, tier 2, and tier 3 [41]. The higher the tier, the higher the level of medical care offered. Tier 3 hospitals are usually large academic centers in major cities [42]. Similarly, anesthesia professionals are categorized by a hierarchical system, including junior (eg, resident and nurse practitioner), intermediate (eg, attending physician and supervisor nurse), deputy senior (eg, associate chief physician and associate chief nurse), and senior (eg, chief physician and chief nurse) [43]. The promotion process for these professional titles is based on a comprehensive evaluation that considers clinical proficiency, years of experience, academic contributions, and formal education [43].

### Knowledge About PAEs

This section assessed the respondents’ knowledge of PAEs, with 12 items consisting of 2 single-choice and 10 true or false questions, based on established medical guidelines, clinical expertise, published articles, and procedural announcements related to PAEs. These sources formed the basis for our scoring criteria.

The questions encompassed various aspects, including the definition, preventability, risks to patients, and the protocols for reporting and managing PAEs. Each question had a definitive correct answer, with binary scoring: 1 point for each correct response, 0 points for incorrect responses, and allowing a maximum score of 12. Higher scores indicate a more comprehensive understanding of PAEs, while lower scores highlight areas where further knowledge enhancement is necessary.

### Attitudes Toward PAEs

Participants’ attitudes toward PAEs and the utilization of a mobile app–based CDSS for managing PAEs were explored in this section, which included 12 questions using a 5-point Likert scale (1=strongly disagree, 2=disagree, 3=neutral, 4=agree, 5=strongly agree). Since this section is subjective, there are no correct or incorrect answers, and the objective is to quantify attitudes. Total scores ranged from 12 to 60, with higher scores indicating a more positive outlook toward the management of PAEs.

### Practices Regarding Perioperative Incident Reporting System

The final segment evaluated participants’ experiences with reporting incidents, along with their perceptions of facilitators and barriers to adopting the mobile app–based CDSS for PAE management. This section included dichotomous and Likert scale questions to provide a comprehensive view of practice behaviors. No formal scoring system was applied, but detailed statistical analyses were performed on response distributions to identify trends and variations in reporting practices. The questionnaire concluded with an optional open-ended question, allowing participants to provide additional insights on PAE management and the proposed system.

### Data Collection

The questionnaire was created and published using Wen Juan Xing, a popular web-based survey platform in China [44]. The survey homepage displayed the objectives, scope, and instructions for participants. A dedicated QR code was generated for easy access and distribution. The director of the Department of Anesthesiology at West China Hospital coordinated with the Chinese Association of Anesthesiologists, which represents anesthesia professionals from most medical institutions nationwide, to endorse and share the survey. With this association’s support, the QR code was distributed through WeChat groups affiliated with anesthesiology departments in various health care organizations, which is a common communication channel within China.

Upon submission, Wen Juan Xing’s backend automatically recorded responses. Given WeChat’s strict real-name verification policy for account creation and Wen Juan Xing’s enforcement of a single submission per unique IP address, it was ensured that each participant used a distinct WeChat account and made only one submission. The platform concealed each participant’s privacy, and no personally identifiable information was collected. Thereafter, data were directly downloaded from Wen Juan Xing to a Microsoft Excel spreadsheet. After excluding duplicate and invalid responses, the organized dataset was prepared for the final analysis.

### Sample Size Estimation

Sample size estimation was based on a previous study surveying a conceptual mobile app–based system for reporting AEs following influenza vaccination, which indicated an 86% acceptance rate among physicians [45]. Using PASS 15 software (NCSS, LLC), we calculated the required sample size as 866, adjusting for a 10% attrition rate, an SD 5% margin of error, and a 95% CI. To reflect the distribution of anesthesiologists and nurse anesthetists in China, which is 76.7% and 23.3% respectively, the final sample consisted of 664 anesthesiologists and 202 nurse anesthetists.

### Statistical Analysis

Data were extracted from the Wen Juan Xing platform and analyzed using SPSS (version 26.0; IBM Corp) and R (version 4.3.1; R Core Team). Continuous variables conforming to a normal distribution were described as means with SDs, while nonnormally distributed variables were presented as medians with IQRs. Categorical variables were expressed as frequencies and percentages. Descriptive statistics, including chi-square tests, Kruskal-Wallis tests, and ANOVA, were applied according to the data characteristics.

The analysis began by examining the correlations between participants’ sociodemographic characteristics, KAP, and the economic development of their regions. A framework analyzing the determinants of KAP was developed through 3 progressively complex models. These models incorporated a broad spectrum of variables, including age, gender, professional role, educational background, professional title, years of service in health care, hospital tier, gross domestic product (GDP) per capita, geographic region, experience with PAEs, use of informatic tools, participation in departmental discussions on PAEs, and satisfaction with current systems.

The analysis proceeded with univariate assessments using linear regression to evaluate individual impacts, followed by multivariate analyses across the 3 adjusted models. Variables were incrementally incorporated from model 1 through model 3, thereby deepening the investigative scope. Model 1 explored the influence of various independent variables on knowledge levels, designated as the dependent variable. Model 2 extended this examination by evaluating the effects of independent variables on attitudes. Ultimately, model 3 used a comprehensive approach, centering on the willingness to use the CDSS as the dependent variable and integrating all previously considered variables. This structured progression facilitated a multifaceted exploration of how knowledge, attitudes, sociodemographic, and economic factors collectively influence the willingness to practice using CDSSs. The significance level was set at *P*<.05.

To mitigate potential responder bias and ensure accurate representation [46], we used a weighted average approach based on the population size of anesthesiologists in each region [47], aiming to assess the willingness of anesthesia providers across various provinces to adopt this mobile app–based CDSS, which served as the main outcome indicator. This method addressed the disproportionate impact of regions with varying anesthesiologist populations on the national average. The approach involved calculating a weighting factor for each province’s reported willingness to use this system, based on its contribution to the total national anesthesiologist population. The formula for the weighting factor is as follows:



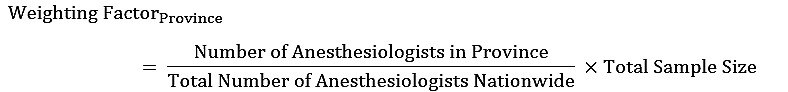



The weighted value was obtained by multiplying the average reported willingness by the calculated weighting factor, ensuring equitable comparison across provinces. This methodology strengthens the reliability and validity of our findings, mitigating biases from unequal data distribution and providing a more accurate national assessment of adoption intentions.

Based on per capita GDP metrics from the National Bureau of Statistics of China for the year 2022, provinces have been classified into 3 economic tiers: high, medium, and low (Multimedia Appendix 3). In addition, this study included 31 mainland provinces, excluding the Hong Kong Special Administrative Region, the Macao Special Administrative Region, and Taiwan Province due to inconsistencies in statistical standards and data collection methodologies. These provinces were grouped into 6 administrative regions based on economic indicators and geographical locations, with further details available from the National Bureau of Statistics website [48].

### Analysis of Open-Ended Questions

Responses were collected in a free-text format, and the data were systematically organized and categorized into key themes based on the frequency of similar comments by 2 independent researchers (XF and PL). Although no formal qualitative analysis was performed, any discrepancies that emerged during the analysis were addressed through discussions with a third researcher (XH).

### Ethical Considerations

The study was approved by the Research Ethics Committee of West China Hospital of Sichuan University (2024-132). Participants were invited to complete the anonymous questionnaire accessed via a QR code, following informed consent. A brief introduction outlining the study’s objectives and privacy assurances was provided to respondents. The survey platform ensured that no personally identifiable information was collected, and all data were deidentified to protect confidentiality. Participants were informed that their responses would remain confidential and used only for research purposes. Participation was entirely voluntary, and participants had the right to withdraw at any time. No compensation was offered for participation.

## Results

### Sociodemographic Characteristics

The survey engaged anesthesia providers throughout China, yielding 2440 valid responses (99.3% of 2456 participants), including 2226 anesthesiologists (91.2%) and 214 nurse anesthetists (8.7%). The median age of respondents was 42 years, with a slight male majority (1307/2440, 51.6%) and fewer females (1133/2440, 46.4%). The distribution of gender and age was consistent across regions, regardless of the GDP per capita. Table 1 provides a descriptive overview of the significant variations observed in the demographics and professional characteristics based on the regional GDP per capita. Notably, the attitudes toward PAEs and willingness to use this mobile app–based CDSS among participants varied in accordance with the GDP per capita of their regions (*P*=.003), while their knowledge levels about this system did not display a similar correlation.

**Table 1:** Demographic characteristics of the participants.

Characteristics		Total (N=2440)	GDP per capita^a^			*χ*^2^ (*df*)	*P* value
			Low (n=668)	Middle (n=904)	High (n=868)		
Age, median (IQR), (%)		42 (15)	42 (17)	42 (14)	41 (14)	1.7802	.41^b^
**Gender, n (%)**						4.9999 (2)	.08^c^
	Men	1307 (53.6)	352 (52.7)	510 (56.4)	445 (51.3)		
	Women	1133 (46.4)	316 (47.3)	394 (43.6)	423 (48.7)		
**Professional role, n (%)**						65.545 (2)	＜.001^c^
	Anesthesiologist	2226 (91.2)	559 (83.7)	852 (94.3)	815 (93.9)		
	Nurse anesthetist	214 (8.8)	109 (16.3)	52 (5.8)	53 (6.1)		
**Tiers of hospital, n (%)**						16.786 (4)	.002^c^
	Tier 1	22 (0.9)	3 (0.5)	13 (1.4)	6 (0.7)		
	Tier 2	538 (22.1)	162 (24.3)	218 (24.1)	158 (18.2)		
	Tier 3	1880 (77.1)	503 (75.3)	673 (74.5)	704 (81.1)		
**Education, n (%)**						93.677 (6)	＜.001^c^
	Junior college or below	25 (1.0)	15 (2.3)	8 (0.9)	2 (0.2)		
	Undergraduate degree	1701 (69.7)	516 (77.3)	667 (73.8)	518 (59.7)		
	Master’s degree	567 (23.2)	111 (16.6)	176 (19.5)	280 (32.3)		
	Doctor’s degree or above	147 (6.0)	26 (3.9)	53 (5.9)	68 (7.9)		
**Professional title, n (%)**						49.569 (6)	<.001^c^
	Junior	438 (18.0)	167 (25.0)	142 (15.7)	129 (14.9)		
	Intermediate	802 (32.9)	175 (26.2)	293 (32.4)	334 (38.5)		
	Deputy senior	667 (27.3)	166 (24.9)	266 (29.4)	235 (27.1)		
	Senior	533 (21.8)	160 (24.0)	203 (22.5)	170 (19.6)		
**Years in practice, n (%)**						5.75 (6)	.45^c^
	≤5	317 (13.0)	97 (14.5)	109 (12.1)	111 (12.8)		
	6-10	381 (15.6)	104 (15.6)	134 (14.8)	143 (16.5)		
	11-19	683 (28.0)	184 (27.5)	245 (27.1)	254 (29.3)		
	≥20	1059 (43.4)	283 (42.4)	416 (46.0)	360 (41.5)		
Knowledge, median (IQR)^d^		11 (2)	11 (2)	11 (2)	11 (2)	1.2572 (2)	.53^b^
Attitude, median (IQR)^e^		51 (7)	50 (6.5)	51 (6)	51 (7)	13.914 (2)	<.001^b^
Adoption willingness, median (IQR)^f^		4 (1)	4 (1)	4 (1)	4 (1)	11.518 (2)	.003^b^

^a^GDP: gross domestic product; high GDP areas include Beijing, Shanghai, Jiangsu, Fujian, Tianjin, Zhejiang, Guangdong, Inner Mongolia, Hubei, Chongqing, and Shandong; medium economic development is noted in Shaanxi, Shanxi, Anhui, Hunan, Jiangxi, Ningxia, Liaoning, Xinjiang, Sichuan, and Hainan; and regions with lower GDP per capita, such as Henan, Yunnan, Qinghai, Tibet, Hebei, Jilin, Guizhou, Guangxi, Heilongjiang, and Gansu, reflect a less affluent economic status.

^b^Kruskal-Wallis test.

^c^Chi-square test.

^d^ Knowledge: Calculated as the sum of correct responses in the knowledge section (maximum=–12), with each correct answer scored as 1 point. This score reflects participants’ understanding of perioperative adverse events.

^e^Attitude: derived from the sum of responses in the attitude section using a 5-point Likert scale (1=strongly disagree, 5=strongly agree). Higher scores indicate more positive attitudes toward PAEs and the use of a mobile app–based clinical decision support system.

^f^Adoption willingness: based on participants’ responses to question 15 in the practices section, measured on a 5-point Likert scale. Higher scores reflect a greater willingness to adopt the mobile app–based CDSS for PAE management.

### Knowledge, Attitude, and Practice Regarding PAEs

#### Knowledge Regarding PAEs

**Figure 1:**
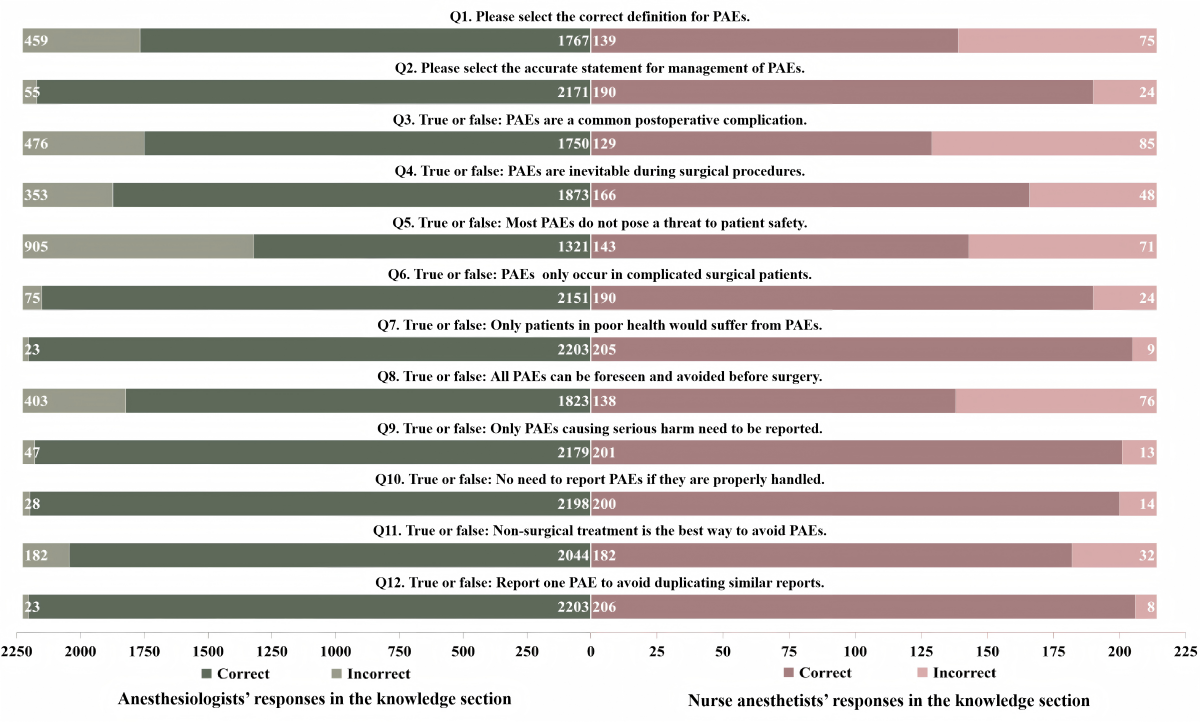
Anesthesia providers' knowledge assessments. The figure includes knowledge responses from nurse anesthetists (right side) and anesthesiologists (left side). CDSS: clinical decision support system; PAE: perioperative adverse event.

Figure 1 illustrates participants’ knowledge regarding PAEs and their management. Anesthesiologists demonstrated slightly higher knowledge scores (mean 10.64, SD 1.37) about PAEs compared to nurse anesthetists (mean 9.76, SD 2.16). Among these participants, 29.7% (662/2226) of anesthesiologists and 12.6% (27/214) of nurse anesthetists answered all questions accurately. Specifically, a significant number of participants, comprising 79.4% (1767/2226) of anesthesiologists and 65% (139/214) of nurse anesthetists, demonstrated a clear understanding definition of PAEs. Moreover, 97.5% (2171/2226) of anesthesiologists and 88.8% (190/214) of nurse anesthetists exhibited a comprehensive grasp of the management goals for PAEs. Unfortunately, 15.9% (353/2226) of anesthesiologists and 22.4% (48/214) of nurse anesthetists were not aware of the preventable nature of PAEs, and out of a total of 2440 anesthesia providers, 976 (40%) overlooked the risks that PAEs pose to patient safety. However, the vast majority acknowledged that PAEs could occur under various conditions, not limited to complex surgeries (2341/2440, 95.9%) or in patients with poor conditions (2408/2440, 98.7%,). In addition, 2380 participants (2380/2440, 97.5%) recognized the importance of reporting instances not only when patients experience serious harm.

#### Attitudes Regarding PAEs

**Figure 2:**
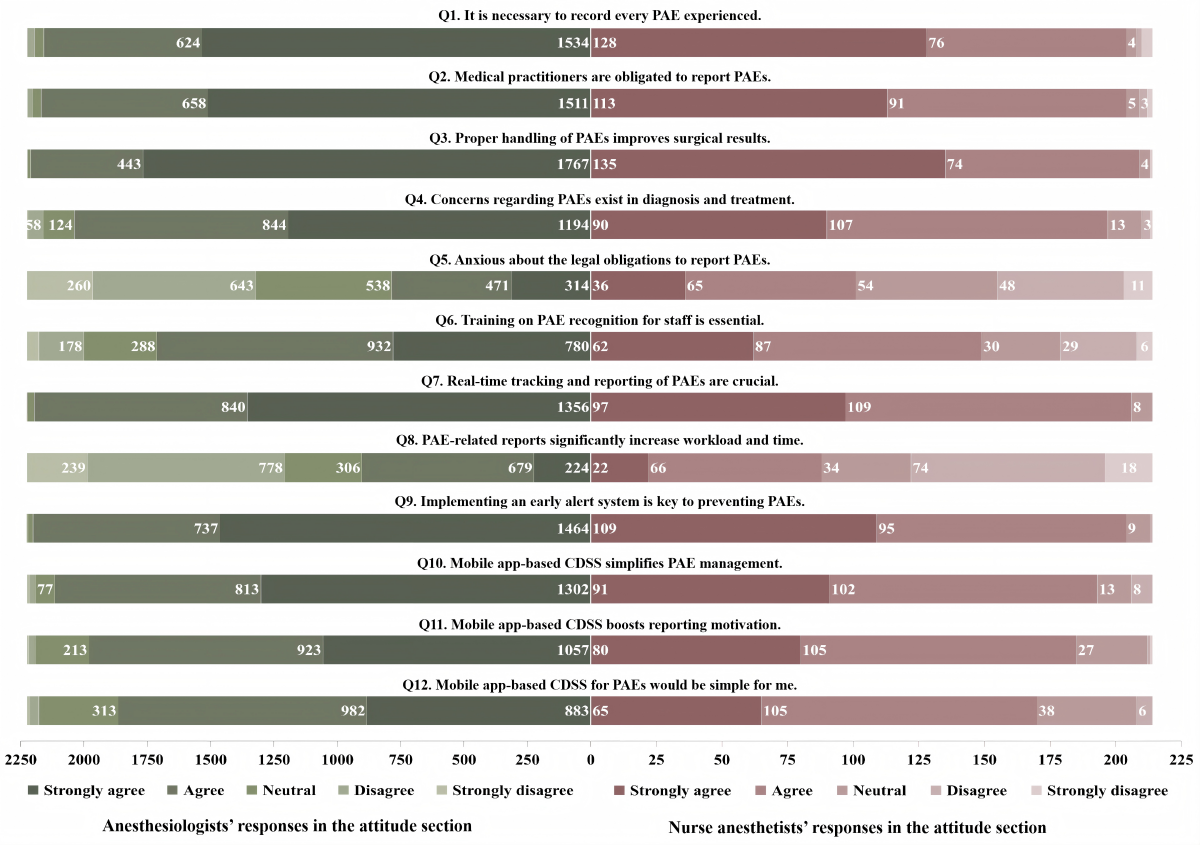
Anesthesia providers’ attitude assessments. The figure includes attitude responses from nurse anesthetists (right side) and anesthesiologists (left side). CDSS: clinical decision support system; PAE: perioperative adverse event.

Figure 2 reveals positive attitudes of anesthesia professionals toward the reporting and management of PAEs, as evidenced by the average scores of anesthesiologists (mean 50.71, SD 4.67) and nurse anesthetists (mean 49.43, SD 5.42). A significant majority (2373/2440, 97.3%), viewed it as a professional responsibility. Despite concerns about impacts on patient outcomes (2235/2440, 91.6%), legal implications (886/2440, 36.3%), and time constraints (991/2440, 40.6%), there was strong support for the adoption of this mobile app–based CDSS to manage PAEs. Specifically, 98.7% (2196/2226) of anesthesiologists and 96.3% (206/214) of nurse anesthetists highlighted the importance of real-time monitoring and reporting. Nearly unanimous support (2405/2440, 98.6%) was observed for the integration of alerts for early detection of PAEs. Furthermore, 94.6% (2308/2440) of anesthesia providers endorsed the mobile app–based CDSS as a tool to improve reporting efficiency, with 88.7% (2165/2440) believing that it could enhance engagement in reporting practices.

#### Practice Experiences Regarding PAEs

Over half (1271/2440, 52.1%) of anesthesia practitioners, including 55.1% (1227/2226) of anesthesiologists and 20.6% (44/214) of nurse anesthetists, encountered PAEs in the last year. Among these, only 650 out of 1271 (51.1%) participants consistently reported every occurrence of incidents. A substantial majority (1940/2440, 79.5%), consisting of 78.2% (1741/2226) of anesthesiologists and 93% (199/214) of nurse anesthetists, reported that their departments regularly hold discussions about PAEs, with a significant number (1849/1940, 95.3%) actively participating in these discussions. Furthermore, out of three-quarters of the respondents whose departments regularly discussed PAEs, 74.8% of anesthesiologists (1302/1741) and 76.9% of nurse anesthetists (153/199), expressed the belief that such discussions could have a positive impact on PAE management.

The majority of participants (1654/2440, 67.8%) did not answer the last open-ended question. For those who did respond, several key themes emerged: A small proportion (92/2440, 3.8%) expressed positive attitudes, including satisfaction, expectations, or support for improving PAE management systems. Many respondents (116/2440, 4.8%) emphasized the importance of user-centered design, advocating for systems that simplify workflows and reduce reporting burdens. Some participants (52/2440, 2.1%) called for strengthened management, highlighting the need for enhanced oversight and systematic approaches to PAEs. A few (41/2440, 1.7%) suggested the establishment of incentive mechanisms, such as reward systems, to encourage incident reporting. Training and education were also highlighted as critical areas, with 1.3% (32/2440) of respondents stressing the importance of programs to improve reporting and management practices. A small number (19/2440, 0.8%) recommended nationwide standardization, including the promotion, popularization, and unification of PAE management systems, particularly in primary hospitals. Concerns about privacy and data security were raised by 0.6% (15/2440) of participants, while 0.5% (13/2440) advocated for responsibility-free reporting systems to foster transparency. Furthermore, 0.4% (10/2440) suggested regular system updates based on users’ feedback, and a minimal number (4/2440, 0.2%) expressed concerns about insufficient funding for research, application, and promotion of PAE management tools. Finally, 3 participants expressed negative attitudes toward the mobile app–based CDSS for PAE management. Specifically, the comments were: “My phone already takes up too much of my time; there’s no need to use an app for reporting,” “The current system works fine; there’s no need to use a new system,” and “No matter what, it still increases the workload of frontline health care workers.”

#### Current Incidents Reporting System Use and Satisfaction

**Figure 3:**
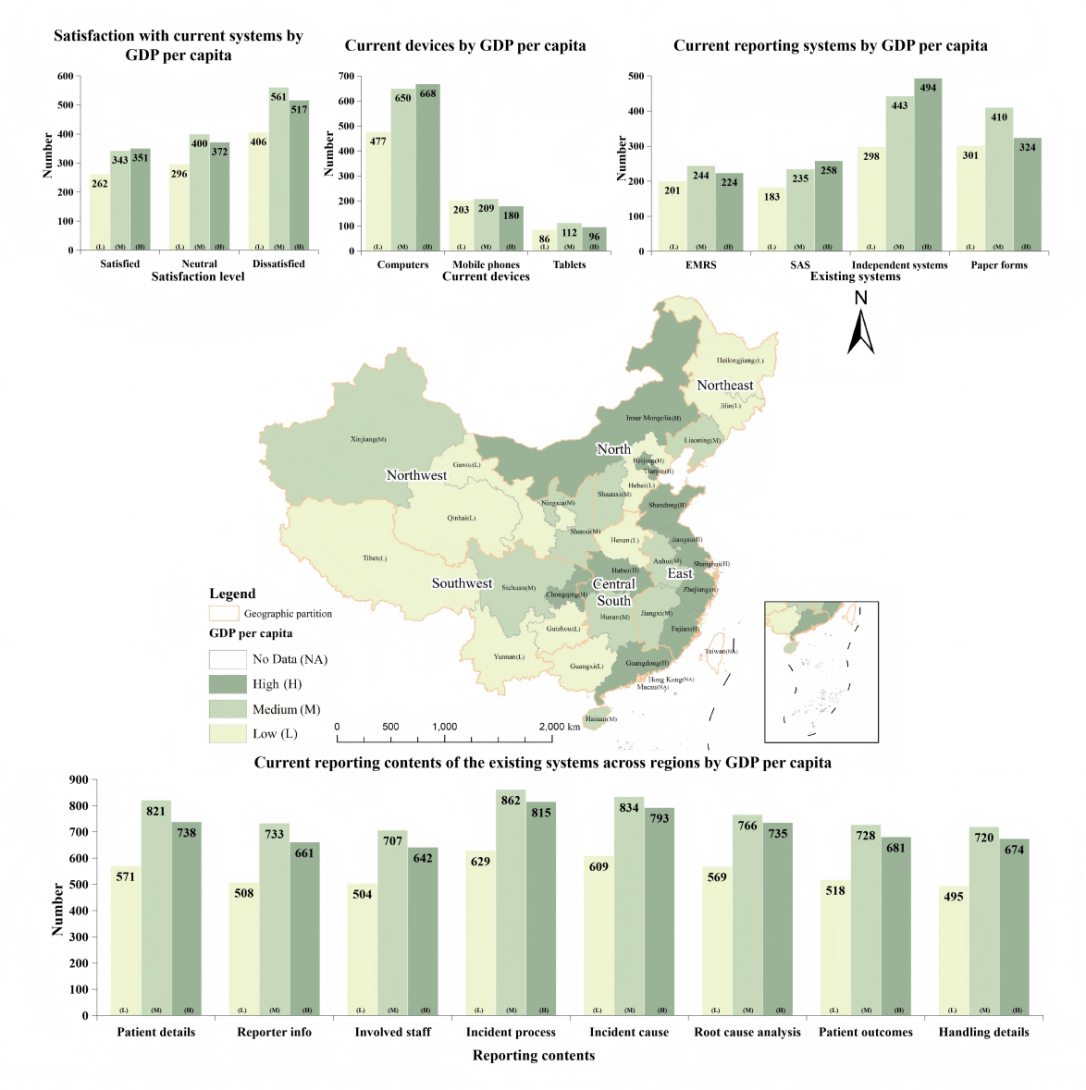
Geographic distribution of provincial GDP per capita tiers in China with associated survey outcomes. The map categorizes China’s mainland provinces into three GDP per capita tiers (low, medium, and high) and presents correlated survey data on (1) participant satisfaction with existing systems, (2) equipment used for PAE management, (3) current system types, and (4) content coverage of current systems. EMRS: electronic medical record systems; GDP: gross domestic product; PAE: perioperative adverse event; SAS: surgical anesthesia systems.

Figure 3 illustrates the functionality of current systems across regions with varying GDP per capita, alongside the satisfaction levels of anesthesiologists and nurse anesthetists toward these systems. Out of the 2440 participants, 77.5% of anesthesia providers (1891/2440) reported using diverse methods to report PAEs. Electronic medical record systems (669/2440, 27.4%), surgical anesthesia systems (676/2440, 27.7%), independent AE reporting systems (1307/2440, 53.6%), and paper forms (1035/2440, 42.4%). Among these anesthesia practitioners with access to structured reporting systems, the predominant access point was through computers (1795/1891, 94.9%), followed by mobile phones (592/1891, 31.3%) and tablets (294/1891, 15.5%). These structured systems were equipped with advanced features including automatic alerts (537/1891, 28.4%), identification mechanisms (591/1891, 31.3%), automated reporting functionalities (528/1891, 27.9%), data retrieval capabilities (824/1891, 43.6%), pop-up reminders (682/1891, 36.1%), and SMS text message notifications (417/1891, 22.1%). Current PAE reporting required capturing comprehensive information encompassing patient demographics (2130/2440, 87.3%), the identity of the reporter (1902/2440, 78%), involved medical professionals (1853/2440, 75.9%), details of the incident process (2306/2440, 94.5%) and its cause (2239/2440, 91.8%), root cause analysis (2070/2440, 84.8%), patient outcomes (1927/2440, 79%), and incident handling specifics (1889/2440, 77.4%). Despite the technological advancements and the array of features these systems offered, only 39.2% (956/2440) of respondents, comprising 828 anesthesiologists and 128 nurse anesthetists, expressed satisfaction with the existing incidents reporting processes.

#### Barriers and Enablers to Adoption of a Mobile App–Based CDSS for PAE Management

**Figure 4:**
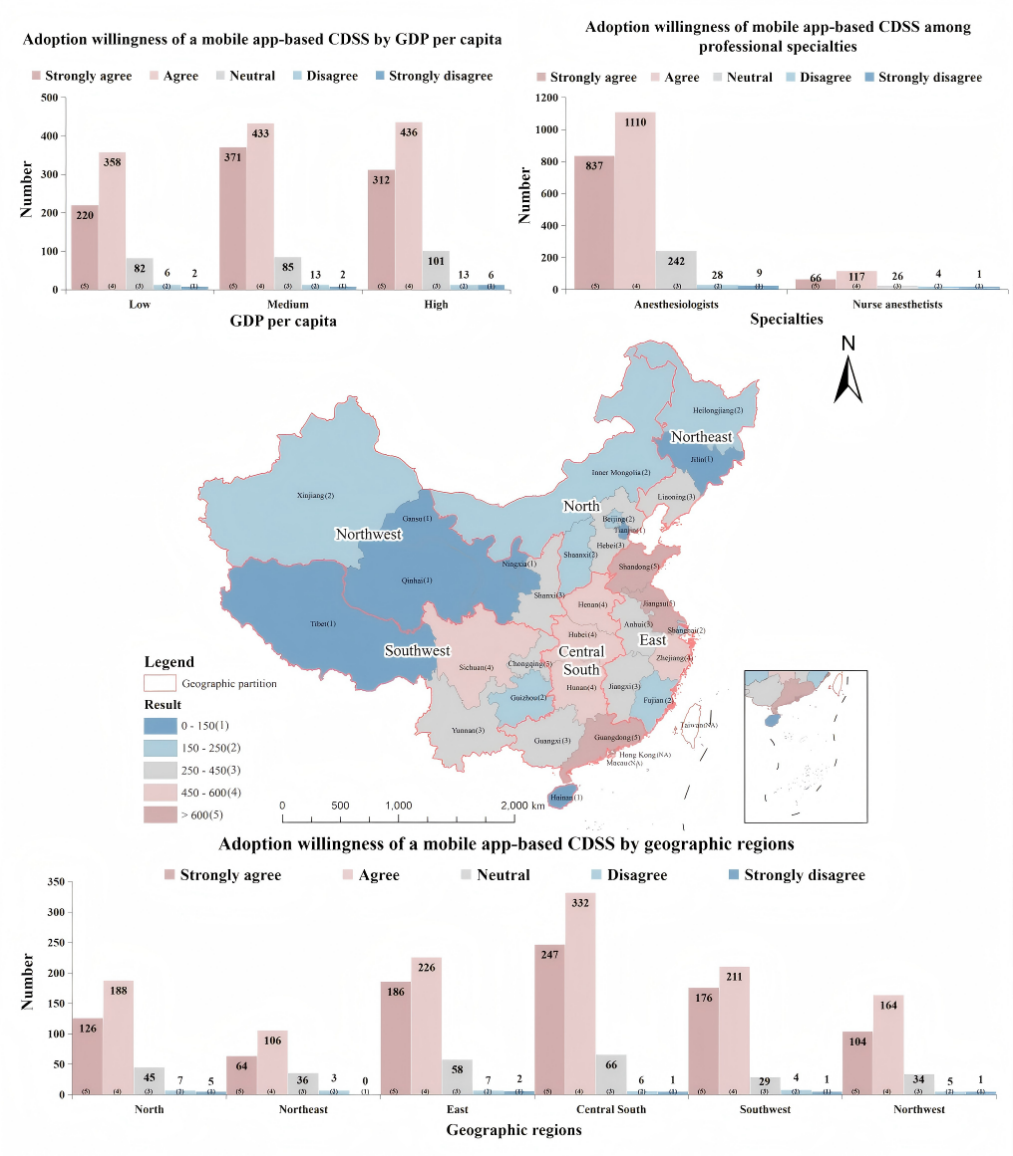
Provincial variation in CDSS adoption willingness across China. The figure shows provincial-level willingness with correlated survey data on adoption attitudes stratified by (1) GDP per capita tiers. (2) professional specialties, and (3) geographic regions. CDSS: clinical decision support system; GDP: gross domestic product.

Figure 4 demonstrates the varying degrees of willingness among anesthesiologists from different provinces to adopt this mobile app***–***based CDSS for managing PAEs. Despite only 28.3% (691/2440) of respondents having previous experience with such digital tools for incident management, a significant 72.1% (498/691) reported positive user experiences. The survey revealed that 87.3% (2130/2440) of participants, comprising 87.5% (1947/2226) anesthesiologists and 85.5% (183/214) nurse anesthetists, are open to using this system for PAE management, as evidenced by the average scores of anesthesiologists (mean 4.23, SD 0.72) and nurse anesthetists (mean 4.14, SD 0.73). Respondents from regions with medium GDP per capita showed the strongest willingness to adopt, representing 33% (804/2440) of the total. Furthermore, participants from the central south region also demonstrated significant interest, making up 23.7% (579/2440) of the total responses.

This map organizes the Chinese mainland into 3 tiers based on GDP per capita, using a color-coding system to distinguish these tiers, with dark green indicating regions of high GDP per capita and light green signifying those with low GDP per capita. In addition, it divides the territory into 6 geographical regions, each outlined with contour lines. These regions are northern (Beijing, Tianjin, Hebei, Shanxi, and Inner Mongolia), northeastern (Liaoning, Jilin, and Heilongjiang), eastern (Shanghai, Jiangsu, Zhejiang, Anhui, Fujian, Jiangxi, and Shandong), central south (Henan, Hubei, Hunan, Guangdong, Guangxi, and Hainan), southwestern (Sichuan, Guizhou, Chongqing, Yunnan, and Tibet), and northwestern (Shaanxi, Gansu, Xinjiang, Qinghai, and Ningxia). Above the map, a series of bar charts are presented, which, from left to right, illustrate the current satisfaction levels of participants with existing systems, the equipment used for managing PAEs, and the forms of these existing systems, all categorized by different levels of GDP per capita. Below the map, another set of bar charts is featured, showcasing the content covered by the current PAE systems across various GDP per capita levels.

The middle map illustrates the varying levels of willingness among provinces to use a CDSS-based mobile app, quantified through a weighted average (weighted results=average willingness of each province×weighting factor). Surrounding auxiliary charts use 5 Likert measures for additional insights. Willingness is color-coded: pink signifies positive adoption willingness, blue indicates reluctance, and gray denotes neutrality. The intensity of each color correlates with the degree of willingness or reluctance, where darker shades represent stronger sentiments towards the mobile app’s adoption. Above the map, 2 bar charts depict the distribution of participants’ willingness to use the CDSS across different per capita GDP regions and various specialties, while the lower bar chart illustrates the distribution of participants’ willingness in different geographic regions.

**Figure 5:**
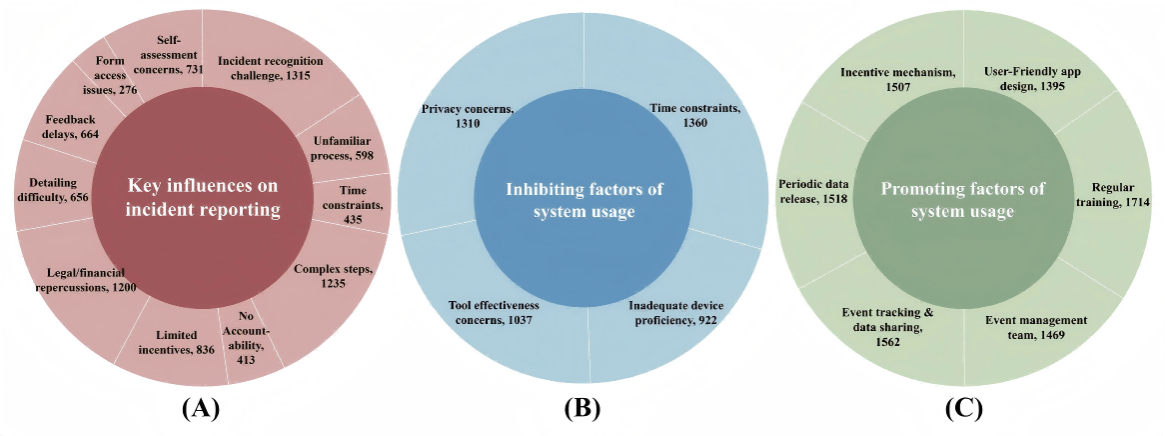
Survey findings on PAE reporting and CDSS adoption, including key influences on PAE reporting and barriers and enablers to using mobile app–based CDSSs. CDSS: clinical decision support system; PAE: perioperative adverse event.

Figure 5 sheds light on the crucial factors influencing incident reporting and identifies several elements that could either foster or hinder interest in this technological innovation. The primary challenges identified in affecting reporting efficacy included the difficulty in recognizing PAEs (1315/2440, 53.9%), the complexity of reporting procedures (1235/2440, 50.6%), the fear of legal and financial repercussions (1200/2440, 49.2%), and the lack of sufficient incentives for reporting (836/2440, 34.3%).To facilitate the adoption of the designed mobile app–based CDSS for PAE management, recommendations included designing an intuitive application interface (1395/2440, 57.2%), providing specialized training (1469/2440, 60.2%), establishing dedicated application management teams (1507/2440, 61.8%), integrating continuous tracking features (1518/2440, 62.2%), consistently sharing reporting data (1562/2440, 64%), and introducing incentive schemes to encourage usage (1714/2440, 70.2%). However, potential barriers to widespread acceptance identified in the survey were limited proficiency with digital devices (922/2440, 37.8%), skepticism about the application’s effectiveness (1037/2440, 42.5%), worries about privacy and security (1310/2440, 53.7%), and concerns that the app usage requires too much time (1360/2440, 55.7%).

#### Correlations Between Knowledge, Attitude, and Practice of PAEs

**Figure 6:**
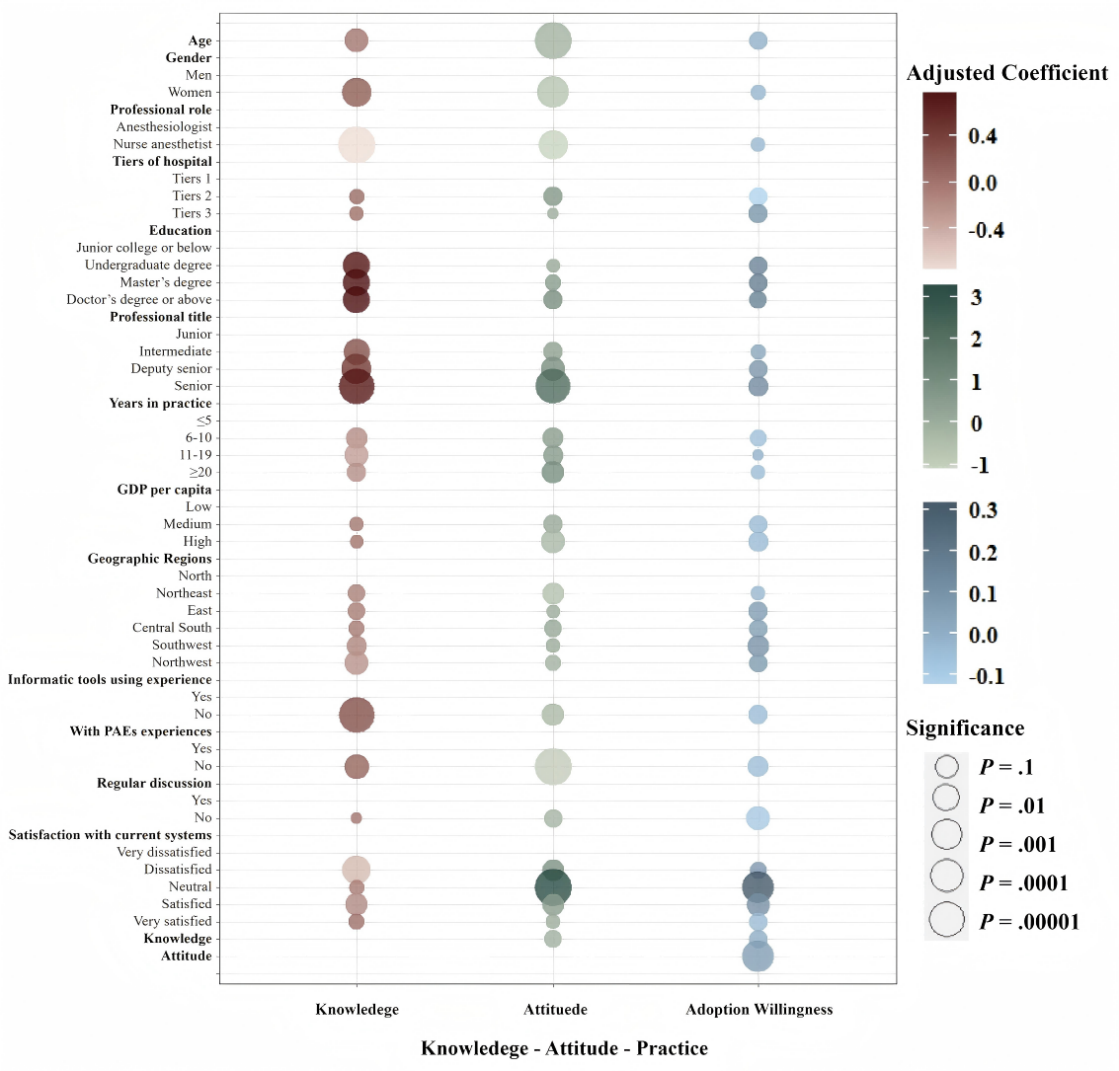
Bubble plot visualizing multivariate regression analysis of the knowledge-attitudes-practices survey on PAEs. CDSS: clinical decision support system; PAE: perioperative adverse event.

Figure 6 and Multimedia Appendix 4 (univariate and multivariate regression analysis of KAP among all participants) present univariate and multivariate regression analysis of knowledge, attitude, and practice willingness among all participants, revealing significant correlations between knowledge, attitude, practice willingness, and various factors. Females (coefficient=0.19, *P*=.003) and individuals with higher educational achievements and professional titles exhibited elevated levels of knowledge, as did respondents lacking experience with similar informatics tools (coefficient=0.29, *P*<.001). In stark contrast, nurse anesthetists (coefficient=–0.76, *P*<.001) and those dissatisfied with current systems (coefficient=–0.51, *P*=.007) demonstrated lower levels of awareness. Interestingly, the assessment of attitudes toward perioperative incident management showed considerable variation across different demographic groups. Older individuals (coefficient=–0.13, *P*<.001), women (coefficient=–0.66, *P*<.001), nurse anesthetists (coefficient=–1.12, *P*=.003), and respondents without previous incidents encounters (coefficient =–0.97, *P*<.001) exhibited more negative attitudes. Conversely, senior professionals (coefficient=2.16, *P*<.001) and those who were neutral toward existing systems (coefficient=3.25, *P*<.001) displayed significantly more positive attitudes toward the reporting and management of PAEs.

In the bubble chart, the color and size of each bubble are used to represent the direction and statistical significance of the impact on knowledge, attitudes, and willingness to adopt new practices. Dark-colored bubbles indicate a positive impact, while light-colored bubbles indicate a negative impact. The size of each bubble corresponds to statistical significance, with larger bubbles representing more significant findings, quantified by the negative logarithm (base 10) of the *P* value. The chart categorizes bubble sizes into 5 significance levels, ranging from a *P* value of .1 (least significant) to 0.00001 (most significant).

**Figure 7:**
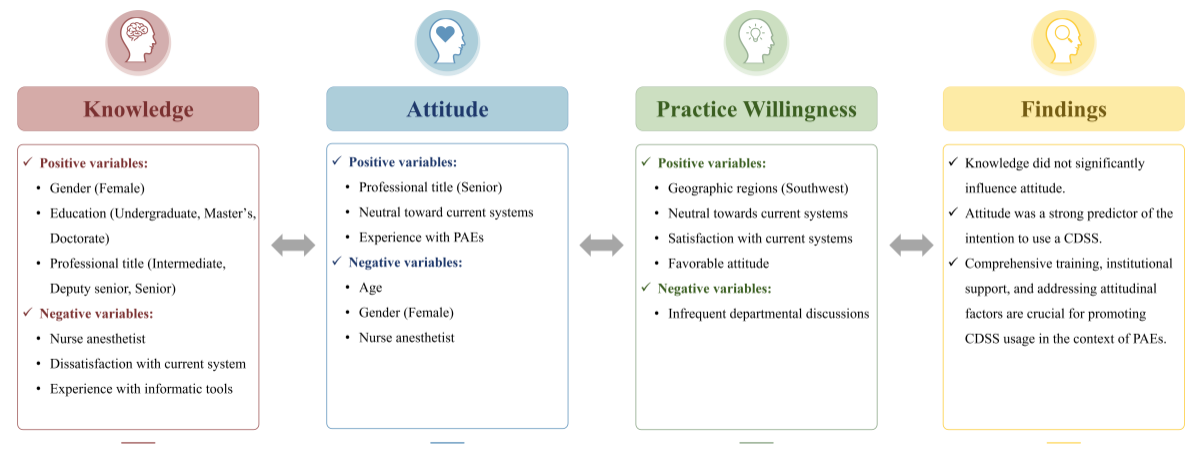
Conceptual framework of factors influencing anesthesia providers’ knowledge acquisition, attitudinal development, and behavioral adoption of CDSSs for PAE management. CDSS: clinical decision support system; PAE: perioperative adverse event.

This study explored the determinants of anesthesia providers’ willingness to implement this mobile app–based CDSS for the management of PAEs. The univariable analysis identified several negative factors associated with willingness to adopt the technology: female gender, longer medical practice tenure, absence of previous incident experience, unfamiliarity with similar informatics tools, and infrequent departmental discussions about PAEs. Positive factors included working in tertiary care (tier 3) hospitals, higher educational qualifications, residing in central southern and southwestern China, and a positive perception of incident management. Multivariate analysis, adjusting for these variables, revealed detailed insights. Anesthesia practitioners in southwest China (coefficient=0.10, *P*<.05), those with a favorable attitude toward managing PAEs (coefficient=0.06, *P*<.001), and individuals neutral (coefficient=0.32, *P*<.001) or satisfied (coefficient=0.11, *P*=.02) about current reporting procedures showed a significantly higher propensity to adopt the innovative system. In contrast, infrequent departmental discussions about PAEs (coefficient=–0.08, *P*=.01) were identified as a negative factor affecting practice willingness. As illustrated in Figure 7, the key determinants influencing participants’ knowledge acquisition, attitudinal orientations, and system adoption willingness are systematically presented.

### Sensitivity Analysis

To enhance the robustness of our findings, a sensitivity analysis was conducted that specifically accounted for the perspectives of anesthesia nurses (Multimedia Appendix 5). Regarding the level of knowledge, those holding deputy senior professional titles exhibited greater awareness (coefficient=1.86, *P*=.04). However, nurse anesthetists used in tier 3 hospitals (coefficient=–1.18, *P*=.04) and residing in the northwestern region of the country (coefficient=–1.09, *P*=.04) demonstrated lower knowledge levels. Moreover, those with deputy senior professional titles also displayed more negative attitudes (coefficient=–4.79, *P*=.02). In contrast, anesthesia nurses who were neutral (coefficient=5.13, *P*<.001), satisfied (coefficient=2.94, *P*=.007), and very satisfied (coefficient=1.46, *P*=.03) with the current reporting system held more positive attitudes toward incidents management, in comparison to those who were dissatisfied. Furthermore, the characteristics associated with a high willingness to adopt among nurse anesthetists included residing in southwest (coefficient=0.43, *P*=.002) and northwest China (coefficient=0.47, *P*=.002), satisfaction with the current system (coefficient=0.37, *P*=.006), and a positive attitude toward PAEs and their management (coefficient=0.05, *P*<.001). Complete details of the survey responses are available in Multimedia Appendix 6.

## Discussion

### Principal Findings

This study is the first comprehensive investigation based on KAP theory regarding PAEs among anesthesia providers in China, which identified key factors influencing the adoption of a mobile app–based CDSS in perioperative care. The high response rate underscores the importance of PAE management and a collective willingness to embrace technological innovations for enhancing patient safety. Many respondents showed a solid understanding and positive attitudes toward PAE management, and a strong willingness to use this informatics solution. These findings are consistent with previous studies in various medical fields, highlighting the increasing acceptance and benefits of integrating informatics tools in health care [49,50].

### Insights from KAP Theory

Grounded in the KAP theory, which elucidates the interrelationships among knowledge, attitudes, and practices in health behavior change [31,51], our study offers valuable insights into the factors influencing the intention to use the CDSS for PAE management among anesthesia providers. This theory posits that behavior change occurs in 3 stages: knowledge accumulation, attitude formation, and behavior promotion, and this framework suggests a positive correlation where knowledge enhances attitudes, which in turn promotes practice [31]. However, our findings revealed a notable divergence: while participants exhibited adequate knowledge regarding PAEs, this knowledge did not significantly influence their attitudes; attitude itself emerged as a strong predictor of the intention to use the CDSS. This suggests, that despite sufficient knowledge, it is participants’ attitudes (shaped by perceived usefulness, ease of use, and trust in the technology), that primarily drive their intention to adopt the CDSS. Therefore, addressing attitudinal factors through targeted interventions is paramount. Comprehensive training and institutional support can cultivate positive attitudes toward the CDSS, ultimately enhancing its integration into clinical practice. In addition, the usability and interface design of this app are critical for its adoption; an unintuitive design might hinder user engagement. Other potential barriers include concerns regarding data privacy and security, resistance to change, and insufficient feedback mechanisms for continuous improvement. An organizational culture that does not encourage innovation, along with the financial implications of implementing such tools, can further restrict adoption. Consequently, understanding these multifaceted factors is crucial for developing effective strategies to promote the adoption of a mobile CDSS.

### Economic Context and Regional Disparities

Building on these findings, it’s essential to consider the broader economic context influencing attitudes toward advanced medical technologies like the mobile app–based CDSS. Despite being the world’s second-largest economy, China’s perioperative medical services lag behind those of developed countries, with notable regional disparities in resource allocation [52,53]. Our study highlighted that staff from tier 3 hospitals, often located in wealthier regions with higher per capita GDP, typically possess higher academic credentials and professional ranks. Interestingly, knowledge about PAEs was consistent across diverse economic zones. However, anesthesia providers in affluent areas demonstrated a stronger inclination to adopt the CDSS, reflecting the influence of regional economic differences on technology perception and accessibility. This openness is likely driven by better financial support and prioritization of perioperative informatics infrastructure in these areas. Wealthier regions, with more comprehensive medical resources, tend to place greater emphasis on these aspects [54]. Furthermore, anesthesiologists in southwest China and nurse anesthetists in western China displayed greater openness to adopting CDSS technology. Despite rapid technological growth, these regions still lag in information technology infrastructure and health care resources compared to more developed eastern provinces [55]. These disparities underscore the need for targeted strategies to bridge these gaps. It is essential to increase financial investments in the perioperative health care sector, offer policy incentives, and enhance medical publicity campaigns [56,57]. Such measures aim to reduce the disparities within China and align its perioperative medical services more closely with those in developed countries.

### Clinical Implications of Gender, Education, and Experience

The analysis of gender disparities suggests that female professionals possess a deeper understanding of PAEs, whereas male professionals exhibit a more positive attitude toward these events and a greater inclination toward adopting this mobile app–based CDSS. This variance might be influenced by gender-specific traits, educational backgrounds, and career paths [49]. Literature suggests women often engage in meticulous learning and adopt a cautious clinical approach, while men display confidence and decisiveness, fostering more optimistic attitudes toward the management of PAEs [58]. These insights warrant further exploration into how gender impacts professional development and service provision for PAEs. Emphasizing gender equity and appreciating diverse perspectives in informatics technology development can lead to more inclusive workplace environments and equitable implementation of technological solutions in the PAE sector [19,49]. Regarding work experience and educational background, the study indicates that higher education levels and professional designations correlate with a better understanding of PAEs. Interestingly, the duration of clinical practice does not significantly enhance the knowledge of PAEs, likely due to China’s health care system, which emphasizes both substantial patient care and scientific research capabilities for career advancement [43]. As physicians gain experience, they often become autonomous and confident [59], potentially reducing their focus on PAEs. Departmental management committees should recognize this trend and promote a culture that values continuous education and prioritizes patient safety PAE management.

In clinical practice, approximately half of the respondents experienced PAEs in the past year, yet nearly 50% of these incidents went unreported. This underreporting is exacerbated by the reliance on voluntary reporting and the absence of effective identification tools [60]. Less than 40% of anesthesia professionals are satisfied with the current incident reporting system, citing its complexity, burdensome workload, and time constraints as major issues. Concerns about new information technology solutions include potential workload increases and data security, reflecting apprehensions noted in previous studies [61]. To address these challenges, an innovative informatics solution could leverage the Institute for Healthcare Improvement’s global trigger tool, proven effective in managing ADEs [62]. As outlined in our early protocol [21], this proposed application should feature real-time monitoring, risk prediction, alerts, and enhanced support for reporting and medical decision-making. Barriers to the current reporting framework include legal and financial concerns, fear of negative personal evaluations, and a lack of reporting incentives. Effective implementation of an intelligent decision-support system for PAEs requires fostering an environment of openness, transparency, collaboration, consistent training, and clear legal protections [61,63]. Such a climate is crucial for maximizing the system’s potential in perioperative care [64,65].

### Perception Differences Among Professional Roles

In addition, this analysis highlighted differences in perceptions between anesthesiologists and nurse anesthetists regarding PAEs. Anesthesiologists generally possessed a deeper understanding and a more positive attitude toward PAE management and were more inclined to adopt the CDSS for this purpose. In contrast, nurse anesthetists demonstrated higher satisfaction with the current incident reporting system. These variations likely stem from the distinct roles and working conditions of the two professions [25]. Anesthesiologists focus on intraoperative patient monitoring, while nurse anesthetists emphasize patient interaction, vigilance, and proactive care, requiring sustained bedside presence and attentiveness beyond merely monitoring and executing medical orders [66]. This makes nurse anesthetists critical stakeholders in perioperative care and PAE monitoring. However, their heavy workload and focus on surgical patients might lead to less attention on PAE management [27], which could explain the study’s limited capture of their perspectives. To improve this situation, respondents emphasized the need for a user-friendly, efficient, and streamlined informatics tool for managing PAEs. Enhancing the role of nurse anesthetists in PAE management and providing targeted training and enhanced education for both anesthesiologists and nurse anesthetists are recommended. Such initiatives could be facilitated by health care departments to improve PAE management and ensure comprehensive stakeholder involvement.

### Limitations and Future Research Directions

Our study has several limitations that should be considered in future research. First, the uneven sampling distribution across different provinces led to potential bias, which we attempted to mitigate through regional analysis and result weighting. Future research could address this by using a more balanced geographic sampling strategy. Second, the open web-based distribution of the survey meant we could not calculate an exact response rate. Future studies might benefit from a closed survey system with targeted recruitment to improve sample representativeness. Third, the low participation of nurse anesthetists, possibly due to the slow growth of the anesthetic nursing sector [25], and a national shortage of anesthesia nurses in China [42], limits the generalizability of our findings to this crucial group. Recognizing their potential contribution to PAE management could affirm their role in perioperative care and encourage further engagement. This recognition could initiate a virtuous cycle, where valuing nurse anesthetists leads to increased engagement and professional development, enhanced patient outcomes, and further acknowledgment of their critical importance. Fourth, our respondent pool was primarily from tertiary hospitals, offering limited insights into PAE management in primary care settings. Fifth, despite our efforts to minimize response bias through clear question design, pilot testing, and anonymity, the risk of response and reporting bias remains. In addition, even though the self-developed questionnaire underwent validity and reliability checks, it still carries the potential for reporting bias. Last, as a cross-sectional study, while we identified factors influencing PAE management, we did not provide a detailed management framework. Future research should focus on developing strategies for implementing CDSSs in managing PAEs.

In light of these limitations, future studies should focus on achieving a more balanced sampling distribution, improving the representation of underrepresented groups like anesthesia nurses, and including primary health care institutions. Incorporating a combination of qualitative and quantitative methods can enhance response validation and provide deeper insights into participants’ motivations. Implementing advanced statistical techniques to identify and adjust for biases can enhance the reliability of the findings. In addition, conducting expert focus groups or Delphi surveys could help develop a comprehensive framework for PAE management using CDSSs. Despite these constraints, the survey offers valuable insights for future management of PAEs and the development of related information technologies.

### Conclusions

This national study highlights a strong readiness among Chinese anesthesia professionals to adopt mobile CDSSs for PAE management. However, critical barriers, including role-specific knowledge disparities and ineffective organizational communication, must be addressed to ensure successful implementation. Collaborative efforts among local authorities, health care facilities, anesthesia departments, and technology developers are essential to design and implement tailored strategies. Key recommendations include interdisciplinary training programs to enhance nurse anesthetists’ competencies, institution-level incentives to promote incident reporting, and user-centered CDSS designs that prioritize seamless integration into clinical workflows. These measures are vital for improving perioperative incident reporting systems and ultimately advancing the safety and outcomes of surgical patients.

## Data Availability

All data collected or analyzed during this study are included in this article and its Multimedia Appendices.

## Appendix

Multimedia Appendix 1 The original questionnaire.

Multimedia Appendix 2 The English translated version of the questionnaire.

Multimedia Appendix 3 Ranking of GDP and GDP per capita by province in China for 2022.

Multimedia Appendix 4 Univariate and multivariate regression analysis of knowledge, attitude and practice willingness among all participants.

Multimedia Appendix 5 Univariate and multivariate regression analysis of knowledge, attitude and practice willingness of nurse anesthetists.

Multimedia Appendix 6 The detailed outcomes of the knowledge, attitude and practice assessment.

## Abbreviations

ADE: adverse drug event
AE: adverse event
CDSS: clinical decision support system
CHERRIES: Checklist for Reporting Results of Internet E-Surveys
GDP: gross domestic product
KAP: knowledge-attitude-practice
PAE: perioperative adverse event
