# Supplementary material for: Barriers and Enablers to Using a Mobile App–Based Clinical Decision Support System in Managing Perioperative Adverse Events Among Anesthesia Providers: Cross-Sectional Survey in China
Source: J Med Internet Res. 2025 May 13;27:e60304. doi: 10.2196/60304 (PMC12117274; doi:10.2196/60304)
Supplement: Multimedia Appendix 1 [file jmir_v27i1e60304_app1.docx]

**围术期不良事件信息系统的认知、态度及需求调查**

尊敬的医护人员：

您好！我国2021-2023年《医疗疗质量安全改进目标》将不良事件管理持续作为改进目标之一，同时，《2021-2030年全球患者安全行动计划》提出不良事件信息化管理是改善医疗照护安全性的重点举措之一。在此背景下，本问卷旨在了解当前麻醉医护对围术期不良事件信息系统的相关认知、态度和行为，明晰相关需求，以指导实践。

在开始问卷之前，我们需要获得您的知情同意。本次调查采取匿名作答，题目选项无对错之分，选择您认可的选项即可。感谢您在百忙之中抽出时间完成这份问卷。您的参与将为提升医疗安全与质量贡献一份力量。再次向您表达诚挚的谢意！

全体调查组人员

**第一部分：基本资料**

1. 您的年龄（岁）：_____
2. 您的性别：A.男 B. 女
3. 您所在医院的级别： A. 一级医院 B. 二级医院 C. 三级医院
4. 贵医院所在城市：_____
5. 您的岗位类别：
6. 外科医师岗
7. 内科医师岗
8. 妇儿医师岗
9. 麻醉医师岗
10. 重症医师岗
11. 疼痛医师岗
12. 全科医师岗
13. 医技岗
14. 护理岗
15. 行政管理岗
16. 其他：_____
17. 您的所属科室：_____
18. 您的最高学位：
19. 专科及以下 B.大学本科 C.硕士研究生 D.博士研究生以上
20. 您的职称：
21. 初级 B.中级 C.副高级 D.正高级
22. 您的工作年限（年）：
23. ≤5 B.6-10 C.11-19 D.≥20

**第二部分：认知**

1. 选择题：以下关于围术期不良事件的定义，您认为下列哪项是正确的?

A. 围术期不良事件是由外科医生造成的伤害

B. 围术期不良事件是手术本身的副作用

C. 围术期不良事件是在围术期诊疗中发生的，与手术目的无关的伤害性事件

D. 围术期不良事件只会发生在手术室内

E. 以上所有

1. 选择题：以下对围术期不良事件管理的描述，您觉得正确的是?

A. 围术期不良事件管理的目的是对围术期不良事件的识别、评估和预防

B. 围术期不良事件管理是为了统计围术期不良事件的发生数量，上报医务部

C. 围术期不良事件管理是指对医务人员惩罚的一种工具

D. 围术期不良事件主要由医院管理层负责填写、上报

1. 围术期不良事件是手术常见的并发症。

A. 是 B. 否

1. 围术期不良事件是手术操作过程中不可避免的。

A. 是 B. 否

1. 大部分围术期不良事件并不会对患者安全产生威胁。

A. 是 B. 否

1. 围术期不良事件可能只发生在接受复杂手术的患者中。

A. 是 B. 否

1. 只有身体状况差的患者，才可能发生围术期不良事件。

A. 是 B. 否

1. 所有围术期不良事件在手术开始前，都可以预测、预防。

A. 是 B. 否

1. 只有对患者造成严重伤害的围术期不良事件才需要报告、记录。

A. 是 B. 否

1. 如果对患者进行了妥善处理，此则围术期不良事件无需报告、记录。

A. 是 B. 否

1. 非手术治疗是避免围术期不良事件的最佳方法。

A. 是 B. 否

1. 若围术期不良事件已报告，没有必要报告新发的类似事件。

A. 是 B. 否

**第三部分：态度**

1. 上报遇到的所有不良事件是有必要的。

A.非常同意 B.同意 C.不确定 D.不同意 E.非常不同意

1. 报告围术期不良事件是医务人员的职责。

A.非常同意 B.同意 C.不确定 D.不同意 E.非常不同意

1. 对围术期不良事件的及时报告和有效管理，将有助于改善手术质量和患者预后。

A.非常同意 B.同意 C.不确定 D.不同意 E.非常不同意

1. 诊疗过程中，会担心患者可能发生围术期不良事件。

A.非常同意 B.同意 C.不确定 D.不同意 E.非常不同意

1. 担心围术期不良事件会给上报人带来法律责任问题。

A.非常担心 B.有点担心 C.不确定 D.不太担心 E.完全不担心

1. 围术期不良事件识别及报告培训，应纳入到员工考核中。

A.非常同意 B.同意 C.不确定 D.不同意 E.非常不同意

1. 围术期不良事件应实时监测并及时报告。

A.非常同意 B.同意 C.不确定 D.不同意 E.非常不同意

1. 围术期不良事件报告会增加额外工作量，占用工作时间。

A.非常同意 B.同意 C.不确定 D.不同意 E.非常不同意

1. 开展围术期不良事件的预警，将有助于医务人员识别，提取预防不良事件。

A.非常同意 B.同意 C.不确定 D.不同意 E.非常不同意

1. 通过移动App（如手机、ipad等）进行围术期不良事件报告/管理，会增加不良事件管理的便捷性。

A.非常同意 B.同意 C.不确定 D.不同意 E.非常不同意

1. 如果能通过移动App上报/管理围术期不良事件，将促进我的上报积极性。

A.非常同意 B.同意 C.不确定 D.不同意 E.非常不同意

1. 如果使用移动App报告/管理围术期不良事件，我对软件操作没有太多困难。

A.非常同意 B.同意 C.不确定 D.不同意 E.非常不同意

**第四部分：实践**

1. 在过去一年中，您诊疗过程中是否发生过围术期不良事件?

A.是 B.否

1. 您是否每次都报告了发生的围术期不良事件？（关联第1题A选项）

A.是 B.否

1. **多选题：**您认为影响您报告围术期不良事件的因素是什么？
2. 无法确定是否为围术期不良事件
3. 不知道如何报告
4. 没有时间报告
5. 报告流程复杂
6. 认为这不是我的责任导致的
7. 缺乏奖励激励上报
8. 担心涉及医疗纠纷、法律责任以及收入减少
9. 难以获取和报告事件的详细信息
10. 报告后反馈不及时
11. 难以获取报告表单
12. 担心上报后，科室和同事对我的工作能力会有负面评价
13. 其他（请简述）_____________
14. 您是否接受过不良事件相关培训?

A.是 B.否

1. 您所在科室，是否会定期开展不良事件讨论?

A.是 B.否

1. 您参加过不良事件讨论吗？（关联第5题A选项）

A.是 B.否

1. 你所在科室，不良事件讨论后会对临床诊疗或管理产生影响（关联第5题A选项）

A.非常同意 B.同意 C.不确定 D.不同意 E.非常不同意

1. **多选题：**您所在科室，当前不良事件上报包含以下哪些内容？

A. 患者信息 B.上报人信息 C.事件相关医护信息 D. 事件发生过程

E. 事件发生原因 F. 事件发生根因分析 G. 患者转归随访结果

H. 事件处理结果 I. 其他（请简述）_____________

1. **多选题：**您所在科室，不良事件报告通过以下哪种形式填报？

A.电子病历系统填报 B.手术麻醉系统填报 C. 独立的不良事件上报系统

D.纸质表格填报 E.其他（请简述）___________

1. **多选题：**此不良事件报告系统，可用的操作客户端包含哪些？（关联第9题ABC选项）

A. 电脑端

B. ipad端

C. 手机端

D. 其他（请简述）_____________

1. **多选题：**此不良事件报告系统，是否涵盖以下功能？（关联第9题ABC选项）

A. 自动预警可能发生的不良事件

B. 识别、抓取可能的不良事件

C. 自动上报识别的可疑不良事件

D. 系统可自动获取电子病历系统中内容，缩短上报时间（如患者信息、手术信息、事件相关医护信息）

E. 事件相关人登录系统时，系统发送弹窗提醒其填报/随访不良事件

F. 系统定时发送短信、电话等提醒至事件相关人手机，提醒其填报/随访不良事件

G. 无

J. 其他（请简述）_____________

1. 您对医院当前不良事件上报流程，是否满意?

A.非常满意 B.满意 C.中立 D不太满意 E.非常不满意

1. 您是否使用过基于信息技术的诊疗途径（如互联网医院、远程问诊、人工智能、3D技术等）？

A.是 B.否

1. 您的体验如何？（关联第13题A选项）

A.非常满意 B.满意 C.一般 D.不满意 E.非常不满意

1. 您是否愿意通过移动App（如手机、ipad等），开展围术期不良事件监测、报告、处理？

A.非常愿意 B.愿意 C.不确定 D.不愿意 E.非常不愿意

1. **多选题：**对于基于移动App的围术期不良事件工具，以下哪些原因可能会阻碍您使用？

A.无法熟练应用智能设备 B.担心此工具的有效性

C.担心个人隐私泄露 D.临床诊疗工作过于繁忙，个人时间有限

E.其他（请简述）_____________

1. **多选题：**对于基于移动App的围术期不良事件管理工具，以下哪些原因可能会促进您使用？

A. 用户友好的App设计

B. 制定激励机制，奖励主动报告人员

C. 提供定期培训

D. 定期发布围术期不良事件相关数据

E. 对发生围术期不良事件的患者进行随访并公布随访数据用于科研分析

F. 组建手术室不良事件管理小组，协调事件相关人员共同上报

G. 其他（请简述）_____________

18. 对于围术期不良事件信息化管理，欢迎您提出宝贵意见和建议：_____________(非必填)
