# Supplementary material for: Barriers and Enablers to Using a Mobile App–Based Clinical Decision Support System in Managing Perioperative Adverse Events Among Anesthesia Providers: Cross-Sectional Survey in China
Source: J Med Internet Res. 2025 May 13;27:e60304. doi: 10.2196/60304 (PMC12117274; doi:10.2196/60304)
Supplement: Multimedia Appendix 2 [file jmir_v27i1e60304_app2.docx]

**Survey on the Knowledge, Attitudes, and Practices of the Perioperative Adverse Events Information System**

Dear Medical Staff,

Greetings! In alignment with China’s 2021-2023 Quality and Safety Improvement Goals for Healthcare, which emphasize the continuous management of adverse events as a pivotal objective, and in light of the 2021-2030 Global Patient Safety Action Plan’s advocacy for enhanced information management of adverse events as a crucial step towards elevating medical care safety, we are conducting a survey. Our goal is to gauge the current knowledge, attitudes, and practices of anesthesiologists and nurse anesthetists concerning perioperative adverse events information systems, to identify specific needs, and to offer actionable guidance.

Before we proceed, we kindly ask for your informed consent to participate in this anonymous survey. We encourage you to select the options that most closely align with your views. Your invaluable input will be instrumental in advancing medical safety and quality.

We greatly appreciate your willingness to dedicate time from your demanding schedule to complete this survey. Your participation is a significant contribution toward the enhancement of medical safety and quality. Once more, we extend our heartfelt thanks to you!

Warm regards,

The Research Team

**Part 1: Basic Information**

1. Age: _____ (*Required)

2. Gender:

A. Male B. Female

3. Tiers of your hospital:

A. Tiers 1 B. Tiers 2 C. Tiers 3

4. City where your hospital is located: _____ (*Required)

5. Your job category:

A. Surgical Physician

B. Internal Medicine Physician

C. Obstetrics and Pediatrics Physician

D. Anesthesiologist

E. Intensive Care Physician

F. Pain Management Physician

G. General Practitioner

H. Medical Technologist

I. Nursing Staff

J. Administrative Staff

K. Other: _____(*Required)

6. Department you belong to: _____(*Required)

7. Highest educational degree:

A. Junior college or below

B. Bachelor’s Degree

C. Master’s Degree

D. Doctoral Degree or Above

8. Professional title:

A. Junior B. Intermediate C. Deputy Senior D. Senior

9. Years of work experience:

A. ≤5 B. 6-10 C. 11-19 D. ≥20

**Part 2: Knowledge**

1. Regarding the definition of perioperative adverse events, which of the following do you think is correct?

A. Perioperative adverse events are injuries caused by surgeons.

B. Perioperative adverse events are side effects of the surgery itself.

C. Perioperative adverse events are harmful incidents that occur during the perioperative care, unrelated to the objective of the surgery.

D. Perioperative adverse events only occur within the operating room.

E. All of the above.

2. Regarding the management of perioperative adverse events, which of the following statements do you think is correct?

A. The purpose of perioperative adverse event management is the identification, assessment, and prevention of perioperative adverse events.

B. Perioperative adverse event management is for counting the number of perioperative adverse events that occur and reporting them to the medical department.

C. Perioperative adverse event management is a tool for punishing medical personnel.

D. Perioperative adverse events are primarily the responsibility of the hospital management to fill out and report.

3. Perioperative adverse events are common complications of surgery.

A. True B. False

4. Perioperative adverse events are inevitable during the surgical procedure.

A. True B. False

5. Most perioperative adverse events do not pose a threat to patient safety.

A. True B. False

6. Perioperative adverse events may only occur in patients undergoing complex surgeries.

A. True B. False

7. Only patients in poor physical condition may experience perioperative adverse events.

A. True B. False

8. All perioperative adverse events can be predicted and prevented before surgery.

A. True B. False

9. Only perioperative adverse events that cause serious harm to patients need to be reported and recorded.

A. True B. False

10. If the patient is properly managed, then the perioperative adverse event does not need to be reported and recorded.

A. True B. False

11. Non-surgical treatment is the best way to avoid perioperative adverse events.

A. True B. False

12. If a perioperative adverse event has been reported, there is no need to report new similar incidents.

A. True B. False

**Part 3: Attitude**

1. It is necessary to report all adverse events encountered.

A. Strongly Agree B. Agree C. Neutral D. Disagree E. Strongly Disagree

2. Reporting perioperative adverse events is the responsibility of medical personnel.

A. Strongly Agree B. Agree C. Neutral D. Disagree E. Strongly Disagree

3. Timely reporting and effective management of perioperative adverse events will help improve surgical quality and patient outcomes.

A. Strongly Agree B. Agree C. Neutral D. Disagree E. Strongly Disagree

4. During the diagnosis and treatment process, there is concern about the potential occurrence of perioperative adverse events.

A. Strongly Agree B. Agree C. Neutral D. Disagree E. Strongly Disagree

5. There is concern that reporting perioperative adverse events could bring legal responsibilities to the reporter.

A. Strongly Agree B. Agree C. Neutral D. Disagree E. Strongly Disagree

6. Training in the identification and reporting of perioperative adverse events should be included in staff assessments.

A. Strongly Agree B. Agree C. Neutral D. Disagree E. Strongly Disagree

7. Perioperative adverse events should be monitored in real-time and reported promptly.

A. Strongly Agree B. Agree C. Neutral D. Disagree E. Strongly Disagree

8. Reporting perioperative adverse events increases additional workload and takes up working time.

A. Strongly Agree B. Agree C. Neutral D. Disagree E. Strongly Disagree

9. The implementation of perioperative adverse event warnings will help medical personnel identify and prevent adverse events.

A. Strongly Agree B. Agree C. Neutral D. Disagree E. Strongly Disagree

10. Reporting/managing perioperative adverse events through a mobile app (such as smartphones, iPads, etc.) will increase the convenience of adverse event management.

A. Strongly Agree B. Agree C. Neutral D. Disagree E. Strongly Disagree

11. If it were possible to report/manage perioperative adverse events through a mobile app, it would increase my motivation to report.

A. Strongly Agree B. Agree C. Neutral D. Disagree E. Strongly Disagree

12. If I were to report/manage perioperative adverse events using a mobile app, I would not have much difficulty operating the software.

A. Strongly Agree B. Agree C. Neutral D. Disagree E. Strongly Disagree

**Part 4: Practice**

1. Have you encountered any perioperative adverse events during your diagnosis and treatment processes in the past year?

A. Yes B. No

2. Have you reported every perioperative adverse event that occurred? (Related to Question 1, Option A)

A. Yes B. No

3. Multiple Choice: What do you think are the factors that affect your reporting of perioperative adverse events?

A. Unable to determine whether it is a perioperative adverse event

B. Not knowing how to report

C. No time to report

D. Reporting process is complicated

E. Believing that it is not my responsibility

F. Lack of rewards to motivate reporting

G. Concerned about involvement in medical disputes, legal responsibilities, and reduced income

H. Difficult to obtain and report detailed information about the event

I. Feedback is not timely after reporting

J. Difficult to obtain reporting forms

K. Concerned that reporting may lead to negative evaluations of my work ability by my department and colleagues

L. Other (Please specify) ______________

4. Have you received training related to adverse events?

A. Yes B. No

5. Does your department regularly conduct discussions on adverse events?

A. Yes B. No

6. Have you participated in discussions on adverse events? (Related to Question 5, Option A)

A. Yes B. No

7. Following discussions on adverse events in your department, there is an impact on clinical diagnosis, treatment, or management (Related to Question 5, Option A)

A. Strongly Agree B. Agree C. Neutral D. Disagree E. Strongly Disagree

8. Multiple Choice: In your department, what does the current adverse event reporting include?

A. Patient information

B. Reporter information

C. Event-related medical and nursing information

D. Event occurrence process

E. Cause of the event

F. Root cause analysis of the event

G. Follow-up results of the patient outcome

H. Event handling results

I. Other (Please specify) ______________

9. Multiple Choice: In your department, how are adverse event reports filled out?

A. Through the electronic medical record system

B. Through the surgery and anesthesia system

C. Through a dedicated adverse event reporting system

D. On paper forms

E. Other (Please specify) _____________

10. Multiple Choice: What are the available operating clients for this adverse event reporting system? (Related to Question 9, Options A, B, C)

A. Computer

B. Tablet

C. Mobile phone

D. Other (Please specify) _______________

11. Multiple Choice: Does this adverse event reporting system include the following features? (Related to Question 9, Options A, B, C)

A. Automatic alerts for potential adverse events

B. Identification and capture of potential adverse events

C. Automatic reporting of identified suspicious adverse events

D. The system can automatically retrieve content from the electronic medical record system to shorten the reporting time (e.g., patient information, surgery information, event-related medical and nursing information)

E. When event-related individuals log into the system, the system sends pop-up reminders to file/follow up on adverse events

F. The system sends periodic reminders via SMS, calls, etc., to the mobile phones of event-related individuals, reminding them to file/follow up on adverse events

G. None

J. Other (Please specify) _______________

12. You are satisfied with the current adverse event reporting process in your hospital.

A. Strongly Agree B. Agree C. Neutral D. Disagree E. Strongly Disagree

13. Have you used IT-based diagnostic and treatment pathways (such as internet hospitals, remote consultations, artificial intelligence, 3D technology, etc.)?

A. Yes B. No

14. How was your experience? (Related to Question 13, Option A)

A. Very Satisfied B. Satisfied C. Neutral D. Dissatisfied E. Very Dissatisfied

15. You are willing to use a mobile app (such as smartphones, iPads, etc.) for monitoring, reporting, and managing perioperative adverse events.

A. Strongly Agree B. Agree C. Neutral D. Disagree E. Strongly Disagree

16. Multiple Choice: Which of the following reasons might hinder your use of a mobile app-based tool for perioperative adverse events?

A. Inability to proficiently use smart devices

B. Concerns about the tool's effectiveness

C. Concerns about personal privacy breaches

D. Being too busy with clinical duties, limited personal time

E. Other (Please specify) _______________

17. Multiple Choice: Which of the following reasons might encourage your use of a mobile app-based management tool for perioperative adverse events?

A. User-friendly app design

B. Implementing incentive mechanisms, rewarding those who report proactively

C. Providing regular training

D. Regularly publishing data related to perioperative adverse events

E. Conducting follow-ups with patients who experienced perioperative adverse events and publishing the follow-up data for research analysis

F. Forming an operating room adverse event management team to coordinate the reporting among involved personnel

G. Other (Please specify) _______________

18. We welcome your valuable suggestions and comments on the information management of perioperative adverse events: _______________ (Optional).
