# Supplementary material for: Barriers and Enablers to Using a Mobile App–Based Clinical Decision Support System in Managing Perioperative Adverse Events Among Anesthesia Providers: Cross-Sectional Survey in China
Source: J Med Internet Res. 2025 May 13;27:e60304. doi: 10.2196/60304 (PMC12117274; doi:10.2196/60304)
Supplement: Multimedia Appendix 3 [file jmir_v27i1e60304_app3.docx]

**Ranking of GDP and Per Capita GDP by Province in China for 2022. ^a^**

|  | **Low** | **Medium** | **High** |
| --- | --- | --- | --- |
| **GDP** | Gansu  Guizhou  Hainan  Heilongjiang  Jilin  Ningxia  Qinghai  Tianjin  Tibet  Xinjiang | Beijing  Chongqing  Guangxi  Hebei  Inner Mongolia  Jiangxi  Liaoning  Shaanxi  Shanxi  Yunnan | Anhui  Fujian  Guangdong  Henan  Hubei  Hunan  Jiangsu  Shandong  Shanghai  Sichuan  Zhejiang |
| **GDP per capita** | Gansu  Guangxi  Guizhou  Hebei  Heilongjiang  Henan  Jilin  Qinghai  Tibet  Yunnan | Anhui  Hainan  Hunan  Jiangxi  Liaoning  Ningxia  Shaanxi  Shanxi  Sichuan  Xinjiang | Beijing  Chongqing  Fujian  Guangdong  Hubei  Inner Mongolia  Jiangsu  Shandong  Shanghai  Tianjin  Zhejiang |

^a^ According to the 2022 data from the National Bureau of Statistics of China, mainland China’s 31 provinces have been categorized into three economic tiers: high, medium, and low. This classification is based on Gross Domestic Product (GDP) and per capita GDP metrics. Regions such as Hong Kong Special Administrative Region (SAR), Macao SAR, and Taiwan Province have been excluded due to different statistical standards and data collection methodologies.
