# Supplementary material for: Barriers and Enablers to Using a Mobile App–Based Clinical Decision Support System in Managing Perioperative Adverse Events Among Anesthesia Providers: Cross-Sectional Survey in China
Source: J Med Internet Res. 2025 May 13;27:e60304. doi: 10.2196/60304 (PMC12117274; doi:10.2196/60304)
Supplement: Multimedia Appendix 4 [file jmir_v27i1e60304_app4.docx]

| Multimedia Appendix 5. Univariate and multivariate regression analysis of knowledge, attitude and practice willingness among all participants. | | | | | | | | | | | | | | | | | | | |
| --- | --- | --- | --- | --- | --- | --- | --- | --- | --- | --- | --- | --- | --- | --- | --- | --- | --- | --- | --- |
|  | | Model 1 - Knowledge | | | | | | Model 2 - Attitude | | | | | | Model 3 - Adoption Willingness | | | | | |
|  | | Unadjusted | | | Adjusted^a^ | | | Unadjusted | | | Adjusted^a^ | | | Unadjusted | | | Adjusted^a^ | | |
|  | | Coefficient | 95% CI^b^ | ***P***-value | Coefficient | 95% CI^b^ | ***P***-value | Coefficient | 95% CI^b^ | ***P***-value | Coefficient | 95% CI^b^ | ***P***-value | Coefficient | 95% CI^b^ | ***P***-value | Coefficient | 95% CI^b^ | ***P***-value |
| Age | | 0.01 | (0.00, 0.01) | .04 | -0.02 | (-0.03, 0.00) | .07 | -0.02 | (-0.04, -0.00) | .049 | -0.13 | (-0.18, -0.08) | <.001 | 0.00 | (-0.01, -0.00) | .03 | 0.00 | (-0.01, 0.00) | .23 |
| **Gender** | |  |  |  |  |  |  |  |  |  |  |  |  |  |  |  |  |  |  |
|  | Men | — | — | — | — | — | — | — | — | — | — | — | — | — | — | — | — | — | — |
|  | Women | 0.07 | (-0.05, 0.19) | .26 | 0.19 | (0.06, 0.31) | .003 | -0.81 | (-1.19, -0.44) | <.001 | -0.66 | (-1.05, -0.27) | <.001 | -0.08 | (-0.14, -0.02) | .006 | -0.02 | (-0.07, 0.04) | .50 |
| **Professional role** | |  |  |  |  |  |  |  |  |  |  |  |  |  |  |  |  |  |  |
|  | Anesthesiologist | — | — | — | — | — | — | — | — | — | — | — | — | — | — | — | — | — | — |
|  | Nurse anesthetist | -0.18 | (-0.22, -0.13) | <.001 | -0.76 | (-0.99, -0.52) | <.001 | -1.27 | (-1.94, -0.61) | <.001 | -1.12 | (-1.86, -0.37) | .003 | -0.09 | (-0.20, 0.01) | .07 | -0.03 | (-0.13, 0.08) | .60 |
| **Tiers of hospital** | |  |  |  |  |  |  |  |  |  |  |  |  |  |  |  |  |  |  |
|  | Tiers 1 | — | — | — | — | — | — | — | — | — | — | — | — | — | — | — | — | — | — |
|  | Tiers 2 | 0.13 | (-0.31, 0.57) | .56 | 0.08 | (-0.35, 0.51) | .71 | 0.81 | (-0.60, 2.22) | .26 | 0.64 | (-0.70, 1.99) | .35 | -0.08 | (-0.29, 0.13) | .47 | -0.12 | (-0.31, 0.07) | .20 |
|  | Tiers 3 | 0.04 | (-0.23, 0.31) | .78 | 0.04 | (-0.23, 0.31) | .77 | 0.35 | (-0.53, 1.22) | .44 | 0.00 | (-0.85, 0.85) | .99 | 0.16 | (0.03, 0.29) | .02 | 0.09 | (-0.03, 0.21) | .13 |
| **Education background** | |  |  |  |  |  |  |  |  |  |  |  |  |  |  |  |  |  |  |
|  | Junior college or below | — | — | — | — | — | — | — | — | — | — | — | — | — | — | — | — | — | — |
|  | Undergraduate degree | 1.34 | (0.76, 1.92) | <.001 | 0.75 | (0.17, 1.34) | .01 | 1.37 | (-0.50, 3.24) | .15 | 0.16 | (-1.68, 1.99) | .87 | 0.28 | (-0.00, 0.57) | .05 | 0.16 | (-0.10, 0.41) | .23 |
|  | Master's degree | 1.59 | (1.00, 2.18) | <.001 | 0.79 | (0.17, 1.40) | .01 | 2.08 | (0.19, 3.98) | .03 | 0.49 | (-1.43, 2.40) | .62 | 0.35 | (0.06, 0.64) | .02 | 0.17 | (-0.09, 0.44) | .20 |
|  | Doctor’s degree or above | 1.61 | (0.99, 2.23) | <.001 | 0.80 | (0.15, 1.45) | .02 | 2.63 | (0.62, 4.64) | .01 | 0.94 | (-1.09, 2.97) | .37 | 0.39 | (0.09, 0.70) | .01 | 0.16 | (-0.12, 0.44) | .26 |
| **Professional title** | |  |  |  |  |  |  |  |  |  |  |  |  |  |  |  |  |  |  |
|  | Junior | — | — | — | — | — | — | — | — | — | — | — | — | — | — | — | — | — | — |
|  | Intermediate | 0.16 | (-0.01, 0.34) | .06 | 0.28 | (0.04, 0.52) | .03 | -0.12 | (-0.67, 0.43) | .67 | 0.40 | (-0.36, 1.15) | .30 | -0.05 | (-0.14, 0.03) | .20 | 0.03 | (-0.07, 0.14) | .53 |
|  | Deputy senior | 0.28 | (0.11, 0.46) | .002 | 0.48 | (0.18, 0.78) | .002 | -0.15 | (-0.72, 0.42) | .61 | 0.90 | (-0.04, 1.85) | .06 | -0.04 | (-0.13, 0.05) | .35 | 0.08 | (-0.05, 0.21) | .21 |
|  | Senior | 0.48 | (0.29, 0.66) | <.001 | 0.72 | (0.38, 1.07) | <.001 | 0.62 | (0.02, 1.22) | .04 | 2.16 | (1.08, 3.25) | <.001 | 0.03 | (-0.06, 0.12) | .50 | 0.13 | (-0.02, 0.28) | .09 |
| **Years in practice** | |  |  |  |  |  |  |  |  |  |  |  |  |  |  |  |  |  |  |
|  | ≤5 | — | — | — | — | — | — | — | — | — | — | — | — | — | — | — | — | — | — |
|  | 6-10 | -0.01 | (-0.23, 0.21) | .92 | -0.18 | (-0.44, 0.08) | .18 | -0.18 | (-0.89, 0.53) | .61 | 0.50 | (-0.33, 1.32) | .24 | -0.12 | (-0.23, -0.01) | .03 | -0.06 | (-0.17, 0.06) | .35 |
|  | 11-19 | -0.06 | (-0.26, 0.13) | .54 | -0.30 | (-0.62, 0.01) | .06 | -0.44 | (-1.07, 0.19) | .17 | 0.56 | (-0.43, 1.55) | .27 | -0.10 | (-0.19, -0.00) | .046 | -0.01 | (-0.15, 0.12) | .85 |
|  | ≥20 | 0.10 | (-0.09, 0.28) | .31 | -0.20 | (-0.62, 0.22) | .35 | -0.45 | (-1.05, 0.15) | .14 | 1.00 | (-0.32, 2.32) | .14 | -0.12 | (-0.21, -0.03) | .007 | -0.05 | (-0.23, 0.14) | .63 |
| **GDP per capita**c | |  |  |  |  |  |  |  |  |  |  |  |  |  |  |  |  |  |  |
|  | Low | — | — | — | — | — | — | — | — | — | — | — | — | — | — | — | — | — | — |
|  | Medium | 0.11 | (0.00, 0.21) | .049 | -0.02 | (-0.15, 0.11) | .76 | 0.51 | (0.18, 0.85) | .003 | 0.18 | (-0.22, 0.58) | .37 | 0.01 | (-0.04, 0.06) | .73 | -0.04 | (-0.09, 0.02) | .20 |
|  | High | 0.00 | (-0.10, 0.09) | .93 | 0.01 | (-0.09, 0.11) | .86 | -0.40 | (-0.72, -0.08) | .01 | -0.30 | (-0.63, 0.03) | .07 | -0.08 | (-0.13, -0.03) | .002 | -0.04 | (-0.08, 0.01) | .11 |
| **Geographic regions** | |  |  |  |  |  |  |  |  |  |  |  |  |  |  |  |  |  |  |
|  | North | — | — | — | — | — | — | — | — | — | — | — | — | — | — | — | — | — | — |
|  | Northeast | 0.06 | (-0.19, 0.31) | .66 | -0.10 | (-0.37, 0.17) | .47 | -0.47 | (-1.27, 0.34) | .25 | -0.59 | (-1.43, 0.26) | .18 | -0.03 | (-0.16, 0.09) | .58 | -0.03 | (-0.14, 0.09) | .67 |
|  | East | -0.10 | (-0.30, 0.10) | .33 | -0.07 | (-0.27, 0.13) | .51 | 0.24 | (-0.40, 0.88) | .46 | 0.05 | (-0.58, 0.68) | .88 | 0.09 | (-0.01, 0.18) | .09 | 0.06 | (-0.03, 0.15) | .17 |
|  | Central South | -0.06 | (-0.25, 0.13) | .54 | -0.05 | (-0.23, 0.14) | .63 | 0.52 | (-0.09, 1.12) | .09 | 0.22 | (-0.37, 0.80) | .47 | 0.11 | (0.02, 0.21) | .01 | 0.05 | (-0.03, 0.14) | .19 |
|  | Southwest | -0.16 | (-0.37, 0.04) | .12 | -0.13 | (-0.36, 0.09) | .25 | 0.39 | (-0.27, 1.06) | .24 | 0.07 | (-0.63, 0.77) | .84 | 0.18 | (0.08, 0.28) | <.001 | 0.10 | (0.00, 0.19) | .048 |
|  | Northwest | -0.35 | (-0.58, -0.13) | .002 | -0.23 | (-0.47, 0.01) | .07 | -0.49 | (-1.21, 0.22) | .18 | -0.15 | (-0.90, 0.61) | .70 | 0.04 | (-0.06, 0.15) | .42 | 0.07 | (-0.04, 0.17) | .20 |
| **With PAEs experiences**^d^ | |  |  |  |  |  |  |  |  |  |  |  |  |  |  |  |  |  |  |
|  | Yes | — | — | — | — | — | — | — | — | — | — | — | — | — | — | — | — | — | — |
|  | No | 0.00 | (-0.12, 0.12) | .99 | 0.12 | (0.00, 0.24) | .05 | -1.22 | (-1.59, -0.84) | <.001 | -0.97 | (-1.35, -0.59) | <.001 | -0.14 | (-0.20, -0.08) | <.001 | -0.05 | (-0.10, 0.00) | .07 |
| **Informatic tools using experience** | |  |  |  |  |  |  |  |  |  |  |  |  |  |  |  |  |  |  |
|  | Yes | — | — | — | — | — | — | — | — | — | — | — | — | — | — | — | — | — | — |
|  | No | 0.42 | (0.29, 0.55) | <.001 | 0.29 | (0.15, 0.42) | <.001 | -1.05 | (-1.46, -0.63) | <.001 | -0.34 | (-0.77, 0.08) | .12 | -0.18 | (-0.24, -0.12) | <.001 | -0.04 | (-0.10, 0.02) | .16 |
| **Regular discussion** | |  |  |  |  |  |  |  |  |  |  |  |  |  |  |  |  |  |  |
|  | Yes | — | — | — | — | — | — | — | — | — | — | — | — | — | — | — | — | — | — |
|  | No | 0.15 | (0.00, 0.29) | .05 | 0.00 | (-0.14, 0.15) | .96 | -0.70 | (-1.17, -0.23) | .003 | -0.20 | (-0.67, 0.27) | .40 | -0.17 | (-0.24, -0.10) | <.001 | -0.08 | (-0.15, -0.02) | .01 |
| **Satisfaction with current systems** | |  |  |  |  |  |  |  |  |  |  |  |  |  |  |  | — | — | — |
|  | Very dissatisfied | — | — | — | — | — | — | — | — | — | — | — | — | — | — | — |  |  |  |
|  | Dissatisfied | -0.7 | (-1.06, -0.34) | <.001 | -0.51 | (-0.88, -0.14) | .007 | 0.73 | (-0.41, 1.87) | .21 | 0.79 | (-0.36, 1.94) | .18 | 0.15 | (-0.02, 0.32) | .09 | 0.08 | (-0.08, 0.24) | .35 |
|  | Neutral | -0.13 | (-0.44, 0.18) | .41 | -0.06 | (-0.37, 0.24) | .68 | 3.35 | (2.37, 4.32) | <.001 | 3.25 | (2.30, 4.21) | <.001 | 0.53 | (0.39, 0.68) | .001 | 0.32 | (0.19, 0.45) | <.001 |
|  | Satisfied | -0.15 | (-0.37, 0.06) | .16 | -0.17 | (-0.37, 0.04) | .12 | 0.70 | (0.04, 1.37) | .04 | 0.46 | (-0.20,1.12) | .17 | 0.17 | (0.07, 0.27) | .001 | 0.11 | (0.02, 0.20) | .02 |
|  | Very satisfied | 0.04 | (-0.09, 0.17) | .54 | 0.03 | (-0.10, 0.16) | .64 | -0.09 | (-0.49, 0.31) | .65 | 0.04 | (-0.35, 0.43) | .84 | -0.05 | (-0.11, 0.01) | .08 | -0.04 | (-0.09, 0.02) | .19 |
| Knowledge | | — | — | — | — | — | — | -0.07 | (-0.20, 0.06) | .28 | -0.05 | (-0.17, 0.08) | .47 | 0.00 | (-0.02, 0.02) | .86 | 0.01 | (-0.01, 0.03) | .19 |
| Attitude | | — | — | — | — | — | — | — | — | — | — | — | — | 0.07 | (0.07, 0.08) | <.001 | 0.06 | (0.06, 0.07) | <.001 |
| aAll models are fully adjusted for all variables listed in the table. Coefficients represent the effect of each variable independently while controlling for the effects of all other variables.  bCI: confidence interval.  cGDP: gross domestic product.  dPAEs: perioperative adverse events. | | | | | | | | | | | | | | | | | | | |
