# Supplementary material for: Barriers and Enablers to Using a Mobile App–Based Clinical Decision Support System in Managing Perioperative Adverse Events Among Anesthesia Providers: Cross-Sectional Survey in China
Source: J Med Internet Res. 2025 May 13;27:e60304. doi: 10.2196/60304 (PMC12117274; doi:10.2196/60304)
Supplement: Multimedia Appendix 5 [file jmir_v27i1e60304_app5.docx]

| Multimedia Appendix 6. Univariate and multivariate regression analysis of knowledge, attitude and practice willingness of nurse anesthetists. | | | | | | | | | | | | | | | | | | | |
| --- | --- | --- | --- | --- | --- | --- | --- | --- | --- | --- | --- | --- | --- | --- | --- | --- | --- | --- | --- |
|  | | Model 1 - Knowledge | | | | | | Model 2 - Attitude | | | | | | Model 3 - Adoption Willingness | | | | | |
|  | | Unadjusted | | | Adjusted^a^ | | | Unadjusted | | | Adjusted^a^ | | | Unadjusted | | | Adjusted^a^ | | |
|  | | Coefficient | 95% CI^b^ | ***P***-value | Coefficient | 95% CI^b^ | ***P***-value | Coefficient | 95% CI^b^ | ***P***-value | Coefficient | 95% CI^b^ | ***P***-value | Coefficient | 95% CI^b^ | ***P***-value | Coefficient | 95% CI^b^ | ***P***-value |
| Age | | 0.02 | (-0.02, 0.06) | .34 | 0.00 | (-0.10, 0.10) | .99 | -0.04 | (-0.14, 0.07) | .49 | 0.09 | (-0.13, 0.31) | .43 | 0.00 | (-0.01, 0.01) | .98 | 0.01 | (-0.02, 0.04) | .47 |
| **Gender** | |  |  |  |  |  |  |  |  |  |  |  |  |  |  |  |  |  |  |
|  | Men | — | — | — | — | — | — | — | — | — | — | — | — | — | — | — | — | — | — |
|  | Women | 0.62 | (-0.29, 1.54) | .18 | 0.43 | (-0.53, 1.40) | .38 | -2.04 | (-4.33, 0.25) | .08 | -1.11 | (-3.34, 1.13) | .33 | -0.32 | (-0.62, -0.01) | .045 | -0.16 | (-0.43, 0.11) | .25 |
| **Tiers of hospital** | |  |  |  |  |  |  |  |  |  |  |  |  |  |  |  |  |  |  |
|  | Tiers 1 | — | — | — | — | — | — | — | — | — | — | — | — | — | — | — | — | — | — |
|  | Tiers 2 | 1.43 | (-0.08, 2.94) | .07 | 1.51 | (-0.04, 3.05) | .06 | -2.80 | (-6.57, 0.98) | .15 | -1.79 | (-5.38, 1.81) | .33 | -0.08 | (-0.59, 0.43) | .76 | 0.17 | (-0.27, 0.60) | .46 |
|  | Tiers 3 | -1.01 | (-2.11, 0.08) | .07 | -1.18 | (-2.30, -0.05) | .04 | 2.82 | (0.07, 5.56) | .05 | 1.73 | (-0.89, 4.35) | .20 | 0.07 | (-0.30, 0.44) | .72 | -0.07 | (-0.39, 0.25) | .67 |
| **Education background** | |  |  |  |  |  |  |  |  |  |  |  |  |  |  |  |  |  |  |
|  | Junior college or below | — | — | — | — | — | — | — | — | — | — | — | — | — | — | — | — | — | — |
|  | Undergraduate degree | 0.35 | (-0.91, 1.62) | .58 | -0.19 | (-1.55, 1.17) | .79 | -0.64 | (-3.80, 2.52) | .69 | 0.12 | (-3.02,3.25) | .94 | -0.21 | (-0.64, 0.21) | .33 | -0.13 | (-0.51,0.25) | .51 |
|  | Master's degree | 1.58 | (-1.66, 4.83) | .34 | 0.10 | (-3.20, 3.40) | .95 | 3.50 | (-4.63, 11.63) | .40 | 5.46 | (-2.15,13.07) | .16 | 0.17 | (-0.93, 1.26) | .77 | 0.32 | (-0.61,1.25) | .50 |
| **Professional title** | |  |  |  |  |  |  |  |  |  |  |  |  |  |  |  |  |  |  |
|  | Junior | — | — | — | — | — | — | — | — | — | — | — | — | — | — | — | — | — | — |
|  | Intermediate | 0.54 | (-0.06, 1.13) | .08 | 0.52 | (-0.31, 1.36) | .22 | -0.15 | (-1.66, 1.37) | .85 | -0.77 | (-2.70,1.16) | .43 | -0.05 | (-0.25, 0.16) | .66 | 0.12 | (-0.12,0.35) | .34 |
|  | Deputy senior | 1.30 | (0.08, 2.53) | .04 | 1.86 | (0.12, 3.60) | .04 | -1.84 | (-4.95, 1.28) | .25 | -4.79 | (-8.85, -0.73) | .02 | 0.08 | (-0.34, 0.50) | .70 | 0.23 | (-0.27,0.73) | .36 |
| **Years in practice** | |  |  |  |  |  |  |  |  |  |  |  |  |  |  |  |  |  |  |
|  | ≤5 | — | — | — | — | — | — | — | — | — | — | — | — | — | — | — | — | — | — |
|  | 6-10 | -0.26 | (-1.04, 0.52) | .51 | -0.58 | (-1.52, 0.36) | .23 | -2.04 | (-3.98, -0.10) | .04 | -1.31 | (-3.49,0.86) | .24 | -0.19 | (-0.45, 0.08) | .16 | -0.08 | (-0.35,0.19) | .55 |
|  | 11-19 | 0.15 | (-0.61, 0.91) | .70 | -0.45 | (-1.77, 0.86) | .50 | -0.68 | (-2.57, 1.21) | .48 | -0.22 | (-3.25,2.82) | .89 | -0.12 | (-0.37, 0.14) | .37 | -0.21 | (-0.58,0.16) | .27 |
|  | ≥20 | 0.33 | (-0.65, 1.30) | .51 | -1.32 | (-3.51, 0.88) | .24 | -1.57 | (-3.99, 0.86) | .21 | -0.33 | (-5.41,4.75) | .90 | -0.09 | (-0.42, 0.23) | .57 | -0.27 | (-0.89,0.34) | .39 |
| **GDP per capita**c | |  |  |  |  |  |  |  |  |  |  |  |  |  |  |  |  |  |  |
|  | Low | — | — | — | — | — | — | — | — | — | — | — | — | — | — | — | — | — | — |
|  | Medium | 0.26 | (-0.24, 0.76) | .30 | -0.01 | (-0.87, 0.86) | .99 | 0.20 | (-1.05, 1.45) | .76 | -0.74 | (-2.74,1.26) | .47 | -0.16 | (-0.32, 0.01) | .06 | 0.21 | (-0.04, 0.45) | .10 |
|  | High | -0.37 | (-0.93, 0.19) | .20 | -0.35 | (-1.04, 0.33) | .31 | -1.36 | (-2.76, 0.04) | .06 | -1.28 | (-2.86, 0.30) | .11 | -0.30 | (-0.48, -0.12) | .002 | 0.02 | (-0.18, 0.21) | .88 |
| **Geographic regions** | |  |  |  |  |  |  |  |  |  |  |  |  |  |  |  |  |  |  |
|  | North | — | — | — | — | — | — | — | — | — | — | — | — | — | — | — | — | — | — |
|  | East | -0.32 | (-1.28, 0.64) | .51 | -0.33 | (-1.65, 0.98) | .62 | 0.65 | (-1.82, 3.11) | .61 | 2.26 | (-0.77, 5.28) | .15 | -0.19 | (-0.51, 0.13) | .24 | -0.36 | (-0.73, 0.01) | .06 |
|  | Central South | 0.54 | (-0.47, 1.56) | .30 | 0.39 | (-0.68, 1.45) | .48 | 1.71 | (-0.90, 4.31) | .20 | 1.54 | (-0.92, 3.99) | .22 | 0.39 | (0.05, 0.72) | .03 | 0.21 | (-0.09, 0.51) | .18 |
|  | Southwest | -0.70 | (-1.57, 0.17) | .12 | -0.54 | (-1.51, 0.43) | .28 | 0.61 | (-1.62, 2.85) | .59 | -0.20 | (-2.44, 2.05) | .86 | 0.39 | (0.10, 0.67) | .009 | 0.43 | (0.15, 0.70) | .002 |
|  | Northwest | -0.95 | (-1.83, -0.06) | .04 | -1.09 | (-2.14, -0.04) | .04 | 0.11 | (-2.17, 2.38) | .93 | -0.04 | (-2.49, 2.41) | .97 | 0.26 | (-0.03, 0.56) | .08 | 0.47 | (0.17, 0.77) | .002 |
| **With PAEs experiences**^d^ | |  |  |  |  |  |  |  |  |  |  |  |  |  |  |  |  |  |  |
|  | Yes | — | — | — | — | — | — | — | — | — | — | — | — | — | — | — | — | — | — |
|  | No | 0.64 | (-0.07, 1.36) | .08 | 0.38 | (-0.35, 1.12) | .31 | -1.40 | (-3.19, 0.39) | .13 | -1.08 | (-2.78, 0.62) | .22 | 0.00 | (-0.24, 0.24) | .99 | 0.15 | (-0.06, 0.36) | .16 |
| **Informatic tools using experience** | |  |  |  |  |  |  |  |  |  |  |  |  |  |  |  |  |  |  |
|  | Yes | — | — | — | — | — | — | — | — | — | — | — | — | — | — | — | — | — | — |
|  | No | 0.78 | (0.20, 1.35) | .009 | 0.59 | (-0.08, 1.25) | .09 | -1.40 | (-2.85, 0.05) | .06 | 0.65 | (-0.89, 2.19) | .41 | -0.34 | (-0.53, -0.15) | <.001 | -0.17 | (-0.36, 0.02) | .08 |
| **Regular discussion** | |  |  |  |  |  |  |  |  |  |  |  |  |  |  |  |  |  |  |
|  | Yes | — | — | — | — | — | — | — | — | — | — | — | — | — | — | — | — | — | — |
|  | No | 0.33 | (-0.81, 1.46) | .57 | 0.11 | (-1.10, 1.32) | .86 | -2.19 | (-5.02, 0.65) | .13 | -1.11 | (-3.90, 1.68) | .44 | -0.29 | (-0.67, 0.09) | .14 | -0.08 | (-0.42, 0.26) | .65 |
| **Satisfaction with current systems** | |  |  |  |  |  |  |  |  |  |  |  |  |  |  |  |  |  |  |
|  | Dissatisfied | — | — | — | — | — | — | — | — | — |  |  |  | — | — | — | — | — | — |
|  | Neutral | -1.33 | (-2.44, -0.22) | .02 | -1.00 | (-2.21, 0.20) | .10 | 4.96 | (2.47, 7.45) | <.001 | 5.13 | (2.34,7.92) | <.001 | 0.68 | (0.35, 1.01) | <.001 | 0.25 | (-0.10, 0.60) | .17 |
|  | Satisfied | -0.10 | (-0.99, 0.78) | .82 | -0.25 | (-1.17, 0.67) | .60 | 3.49 | (1.51, 5.46) | <.001 | 2.94 | (0.82,5.06) | .007 | 0.50 | (0.23, 0.76) | <.001 | 0.37 | (0.11, 0.64) | .006 |
|  | Very satisfied | -0.26 | (-0.83, 0.30) | .36 | -0.18 | (-0.76, 0.40) | .54 | 1.48 | (0.21, 2.74) | .02 | 1.46 | (0.13,2.80) | .03 | 0.07 | (-0.09, 0.24) | .39 | -0.05 | (-0.21, 0.12) | .57 |
| Knowledge | | — | — | — | — | — | — | -0.43 | (-0.76, -0.10) | .01 | -0.21 | (-0.54,0.12) | .21 | -0.04 | (-0.08, 0.01) | .13 | 0.01 | (-0.03, 0.05) | .58 |
| Attitude | | — | — | — | — | — | — | — | — | — | — | — | — | 0.06 | (0.05, 0.08) | <.001 | 0.05 | (0.03, 0.06) | <.001 |
| aAll models are fully adjusted for all variables listed in the table. Coefficients represent the effect of each variable independently while controlling for the effects of all other variables.  bCI: confidence interval.  cGDP: gross domestic product.  dPAEs: perioperative adverse events. | | | | | | | | | | | | | | | | | | | |
