# Supplementary material for: Barriers and Enablers to Using a Mobile App–Based Clinical Decision Support System in Managing Perioperative Adverse Events Among Anesthesia Providers: Cross-Sectional Survey in China
Source: J Med Internet Res. 2025 May 13;27:e60304. doi: 10.2196/60304 (PMC12117274; doi:10.2196/60304)
Supplement: Multimedia Appendix 6 [file jmir_v27i1e60304_app6.docx]

**The detailed outcomes of the knowledge, attitude and practice assessment.**

| **Part 2: Knowledge** | **Number** | **Proportion** |
| --- | --- | --- |
| **1. Regarding the definition of perioperative adverse events, which of the following do you think is correct?** |  |  |
| A. Perioperative adverse events are injuries caused by surgeons. | 10 | 0.4% |
| B. Perioperative adverse events are side effects of the surgery itself. | 29 | 1.2% |
| C. Perioperative adverse events are harmful incidents that occur during the perioperative care, unrelated to the objective of the surgery. | 1906 | 78.1% |
| D. Perioperative adverse events only occur within the operating room. | 5 | 0.2% |
| E. All of the above. | 490 | 20.1% |
| **2. Regarding the management of perioperative adverse events, which of the following statements do you think is correct?** |  |  |
| A. The purpose of perioperative adverse event management is the identification, assessment, and prevention of perioperative adverse events. | 2361 | 96.8% |
| B. Perioperative adverse event management is for counting the number of perioperative adverse events that occur and reporting them to the medical department. | 60 | 2.5% |
| C. Perioperative adverse event management is a tool for punishing medical personnel. | 1 | 0.04% |
| D. Perioperative adverse events are primarily the responsibility of the hospital management to fill out and report. | 18 | 0.7% |
| **3. Perioperative adverse events are common complications of surgery.** |  |  |
| A. True | 561 | 23.0% |
| B. False | 1879 | 77.0% |
| **4. Perioperative adverse events are inevitable during the surgical procedure.** |  |  |
| A. True | 401 | 16.4% |
| B. False | 2039 | 83.6% |
| **5. Most perioperative adverse events do not pose a threat to patient safety.** |  |  |
| A. True | 976 | 40.0% |
| B. False | 1464 | 60.0% |
| **6. Perioperative adverse events may only occur in patients undergoing complex surgeries.** |  |  |
| A. True | 99 | 4.1% |
| B. False | 2341 | 95.9% |
| **7. Only patients in poor physical condition may experience perioperative adverse events.** |  |  |
| A. True | 32 | 1.3% |
| B. False | 2408 | 98.7% |
| **8. All perioperative adverse events can be predicted and prevented before surgery.** |  |  |
| A. True | 479 | 19.6% |
| B. False | 1961 | 80.4% |
| **9. Only perioperative adverse events that cause serious harm to patients need to be reported and recorded.** |  |  |
| A. True | 60 | 2.5% |
| B. False | 2380 | 97.5% |
| **10. If the patient is properly managed, then the perioperative adverse event does not need to be reported and recorded.** |  |  |
| A. True | 42 | 1.7% |
| B. False | 2398 | 98.3% |
| **11. Non-surgical treatment is the best way to avoid perioperative adverse events.** |  |  |
| A. True | 214 | 8.8% |
| B. False | 2226 | 91.2% |
| **12. If a perioperative adverse event has been reported, there is no need to report new similar incidents.** |  |  |
| A. True | 31 | 1.3% |
| B. False | 2409 | 98.7% |
| **Part 3: Attitude** | **Number** | **Proportion** |
| **1. It is necessary to report all adverse events encountered.** |  |  |
| A. Strongly Agree | 1662 | 68.1% |
| B. Agree | 700 | 28.7% |
| C. Neutral | 42 | 1.7% |
| D. Disagree | 28 | 1.2% |
| E. Strongly Disagree | 8 | 0.3% |
| **2. Reporting perioperative adverse events is the responsibility of medical personnel.** |  |  |
| A. Strongly Agree | 1624 | 66.6% |
| B. Agree | 749 | 30.7% |
| C. Neutral | 40 | 1.6% |
| D. Disagree | 23 | 0.9% |
| E. Strongly Disagree | 4 | 0.2% |
| **3. Timely reporting and effective management of perioperative adverse events will help improve surgical quality and patient outcomes.** |  |  |
| A. Strongly Agree | 1902 | 78.0% |
| B. Agree | 517 | 21.2% |
| C. Neutral | 18 | 0.7% |
| D. Disagree | 2 | 0.08% |
| E. Strongly Disagree | 1 | 0.04% |
| **4. During the diagnosis and treatment process, there is concern about the potential occurrence of perioperative adverse events.** |  |  |
| A. Strongly Agree | 1284 | 52.6% |
| B. Agree | 951 | 39.0% |
| C. Neutral | 137 | 5.6% |
| D. Disagree | 61 | 2.5% |
| E. Strongly Disagree | 7 | 0.3% |
| **5. There is concern that reporting perioperative adverse events could bring legal responsibilities to the reporter.** |  |  |
| A. Strongly Agree | 350 | 14.3% |
| B. Agree | 536 | 22.0% |
| C. Neutral | 592 | 24.3% |
| D. Disagree | 691 | 28.3% |
| E. Strongly Disagree | 271 | 11.1% |
| **6. Training in the identification and reporting of perioperative adverse events should be included in staff assessments.** |  |  |
| A. Strongly Agree | 842 | 34.5% |
| B. Agree | 1019 | 41.8% |
| C. Neutral | 318 | 13.0% |
| D. Disagree | 207 | 8.5% |
| E. Strongly Disagree | 54 | 2.2% |
| **7. Perioperative adverse events should be monitored in real-time and reported promptly.** |  |  |
| A. Strongly Agree | 1453 | 59.6% |
| B. Agree | 949 | 38.9% |
| C. Neutral | 35 | 1.4% |
| D. Disagree | 2 | 0.08% |
| E. Strongly Disagree | 1 | 0.04% |
| **8. Reporting perioperative adverse events increases additional workload and takes up working time.** |  |  |
| A. Strongly Agree | 246 | 10.1% |
| B. Agree | 745 | 30.5% |
| C. Neutral | 340 | 13.9% |
| D. Disagree | 852 | 34.9% |
| E. Strongly Disagree | 257 | 10.5% |
| **9. The implementation of perioperative adverse event warnings will help medical personnel identify and prevent adverse events.** |  |  |
| A. Strongly Agree | 1573 | 64.5% |
| B. Agree | 832 | 34.1% |
| C. Neutral | 28 | 1.2% |
| D. Disagree | 6 | 0.3% |
| E. Strongly Disagree | 1 | 0.04% |
| **10. Reporting/managing perioperative adverse events through a mobile app (such as smartphones, iPads, etc.) will increase the convenience of adverse event management.** |  |  |
| A. Strongly Agree | 1393 | 57.1% |
| B. Agree | 915 | 37.5% |
| C. Neutral | 90 | 3.7% |
| D. Disagree | 32 | 1.3% |
| E. Strongly Disagree | 10 | 0.4% |
| **11. If it were possible to report/manage perioperative adverse events through a mobile app, it would increase my motivation to report.** |  |  |
| A. Strongly Agree | 1137 | 46.6% |
| B. Agree | 1028 | 42.1% |
| C. Neutral | 240 | 9.8% |
| D. Disagree | 25 | 1.0% |
| E. Strongly Disagree | 10 | 0.4% |
| **12. If I were to report/manage perioperative adverse events using a mobile app, I would not have much difficulty operating the software.** |  |  |
| A. Strongly Agree | 948 | 38.9% |
| B. Agree | 1087 | 44.5% |
| C. Neutral | 351 | 14.4% |
| D. Disagree | 44 | 1.8% |
| E. Strongly Disagree | 10 | 0.4% |
| **Part 4: Practice** | **Number** | **Proportion** |
| **1. Have you encountered any perioperative adverse events during your diagnosis and treatment processes in the past year?** |  |  |
| A. Yes | 1271 | 52.1% |
| B. No | 1169 | 47.9% |
| **2. Have you reported every perioperative adverse event that occurred? (Related to Question 1, Option A, with a total of 1271 participants)** |  |  |
| A. Yes | 650 | 51.1% |
| B. No | 621 | 48.9% |
| **3. Multiple Choice: What do you think are the factors that affect your reporting of perioperative adverse events?** |  |  |
| A. Unable to determine whether it is a perioperative adverse event | 1315 | 53.9% |
| B. Not knowing how to report | 598 | 24.5% |
| C. No time to report | 435 | 17.8% |
| D. Reporting process is complicated | 1235 | 50.6% |
| E. Believing that it is not my responsibility | 413 | 16.9% |
| F. Lack of rewards to motivate reporting | 836 | 34.3% |
| G. Concerned about involvement in medical disputes, legal responsibilities, and reduced income | 1200 | 49.2% |
| H. Difficult to obtain and report detailed information about the event | 656 | 26.9% |
| I. Feedback is not timely after reporting | 664 | 27.2% |
| J. Difficult to obtain reporting forms | 276 | 11.3% |
| K. Concerned that reporting may lead to negative evaluations of my work ability by my department and colleagues | 731 | 30.0% |
| L. Other (Please specify) ______________ | 28 | 1.2% |
| **4. Have you received training related to adverse events?** |  |  |
| A. Yes | 1697 | 69.5% |
| B. No | 743 | 30.5% |
| **5. Does your department regularly conduct discussions on adverse events?** |  |  |
| A. Yes | 1940 | 79.5% |
| B. No | 500 | 20.5% |
| **6. Have you participated in discussions on adverse events? (Related to Question 5, Option A, with a total of 1940 participants)** |  |  |
| A. Yes | 1849 | 95.3% |
| B. No | 91 | 4.7% |
| **7. Following discussions on adverse events in your department, there is an impact on clinical diagnosis, treatment, or management (Related to Question 5, Option A, with a total of 1940 participants)** |  |  |
| A. Strongly Agree | 616 | 31.8% |
| B. Agree | 839 | 43.2% |
| C. Neutral | 252 | 13.0% |
| D. Disagree | 186 | 9.6% |
| E. Strongly Disagree | 47 | 2.4% |
| **8. Multiple Choice: In your department, what does the current adverse event reporting include?** |  |  |
| A. Patient information | 2130 | 87.3% |
| B. Reporter information | 1902 | 78.0% |
| C. Event-related medical and nursing information | 1853 | 75.9% |
| D. Event occurrence process | 2306 | 94.5% |
| E. Cause of the event | 2239 | 91.8% |
| F. Root cause analysis of the event | 2070 | 84.8% |
| G. Follow-up results of the patient outcome | 1927 | 79.0% |
| H. Event handling results | 1889 | 77.4% |
| I. Other (Please specify) ______________ | 33 | 1.4% |
| **9. Multiple Choice: In your department, how are adverse event reports filled out?** |  |  |
| A. Through the electronic medical record system | 669 | 27.4% |
| B. Through the surgery and anesthesia system | 676 | 27.7% |
| C. Through a dedicated adverse event reporting system | 1307 | 53.6% |
| D. On paper forms | 1035 | 42.4% |
| E. Other (Please specify) _____________ | 51 | 2.1% |
| **10. Multiple Choice: What are the available operating clients for this adverse event reporting system? (Related to Question 9, Options A, B, C, with a total of 1891 participants)** |  |  |
| A. Computer | 1795 | 94.9% |
| B. Tablet | 294 | 15.5% |
| C. Mobile phone | 592 | 31.3% |
| D. Other (Please specify) _______________ | 25 | 1.3% |
| **11. Multiple Choice: Does this adverse event reporting system include the following features? (Related to Question 9, Options A, B, C, with a total of 1891 participants)** |  |  |
| A. Automatic alerts for potential adverse events | 537 | 28.4% |
| B. Identification and capture of potential adverse events | 591 | 31.3% |
| C. Automatic reporting of identified suspicious adverse events | 528 | 27.9% |
| D. The system can automatically retrieve content from the electronic medical record system to shorten the reporting time (e.g., patient information, surgery information, event-related medical and nursing information) | 824 | 43.6% |
| E. When event-related individuals log into the system, the system sends pop-up reminders to file/follow up on adverse events | 682 | 36.1% |
| F. The system sends periodic reminders via SMS, calls, etc., to the mobile phones of event-related individuals, reminding them to file/follow up on adverse events | 417 | 22.1% |
| G. None | 654 | 34.6% |
| J. Other (Please specify) _______________ | 11 | 0.6% |
| **12. You are satisfied with the current adverse event reporting process in your hospital.** |  |  |
| A. Strongly Agree | 220 | 9.0% |
| B. Agree | 736 | 30.2% |
| C. Neutral | 1068 | 43.8% |
| D. Disagree | 387 | 15.9% |
| E. Strongly Disagree | 29 | 1.2% |
| **13. Have you used IT-based diagnostic and treatment pathways (such as internet hospitals, remote consultations, artificial intelligence, 3D technology, etc.)?** |  |  |
| A. Yes | 691 | 28.3% |
| B. No | 1749 | 71.7% |
| **14. How was your experience? (Related to Question 13, Option A, with a total of 691 participants)** |  |  |
| A. Very Satisfied | 175 | 25.3% |
| B. Satisfied | 323 | 46.7% |
| C. Neutral | 190 | 27.5% |
| D. Dissatisfied | 3 | 0.4% |
| E. Very Dissatisfied | 0 | 0.0% |
| **15. You are willing to use a mobile app (such as smartphones, iPads, etc.) for monitoring, reporting, and managing perioperative adverse events.** |  |  |
| A. Strongly Agree | 903 | 37.0% |
| B. Agree | 1227 | 50.3% |
| C. Neutral | 268 | 11.0% |
| D. Disagree | 32 | 1.3% |
| E. Strongly Disagree | 10 | 0.4% |
| **16. Multiple Choice: Which of the following reasons might hinder your use of a mobile app-based tool for perioperative adverse events?** |  |  |
| A. Inability to proficiently use smart devices | 922 | 37.8% |
| B. Concerns about the tool's effectiveness | 1037 | 42.5% |
| C. Concerns about personal privacy breaches | 1310 | 53.7% |
| D. Being too busy with clinical duties, limited personal time | 1360 | 55.7% |
| E. Other (Please specify) _______________ | 41 | 1.7% |
| **17. Multiple Choice: Which of the following reasons might encourage your use of a mobile app-based management tool for perioperative adverse events?** |  |  |
| A. User-friendly app design | 1395 | 57.2% |
| B. Implementing incentive mechanisms, rewarding those who report proactively | 1714 | 70.2% |
| C. Providing regular training | 1469 | 60.2% |
| D. Regularly publishing data related to perioperative adverse events | 1562 | 64.0% |
| E. Conducting follow-ups with patients who experienced perioperative adverse events and publishing the follow-up data for research analysis | 1518 | 62.2% |
| F. Forming an operating room adverse event management team to coordinate the reporting among involved personnel | 1507 | 61.8% |
| G. Other (Please specify) _______________ | 14 | 0.6% |
| **18. We welcome your valuable suggestions and comments on the information management of perioperative adverse events (Optional).** |  |  |
| Not answered. | 1654 | 67.8% |
| No idea. | 415 | 17.0% |
| Positive attitudes: Satisfied / expected / supportive | 92 | 3.8% |
| User-centered design to simplify the workflow | 116 | 4.8% |
| Strengthen the management of perioperative adverse events | 52 | 2.1% |
| Establish a reward mechanism to encourage event reporting | 41 | 1.7% |
| Strengthen training on reporting and management of perioperative adverse events | 32 | 1.3% |
| Suggest nationwide promotion, popularization, and unification, especially in primary hospitals | 19 | 0.8% |
| Protect privacy and data security | 15 | 0.6% |
| Suggest responsibility-free reporting | 13 | 0.5% |
| Regularly update the system based on feedback | 10 | 0.4% |
| Concerns about funding, lack of sufficient funds for research, application, and promotion | 4 | 0.2% |
| Negative attitudes:  1. “My phone already takes up too much of my time; there’s no need to use an app for reporting.”  2. “The current system works fine; there’s no need to use a new system.”  3. “No matter what, it still increases the workload of frontline healthcare workers.” | 3 | 0.1% |
